# An Oncogenic Virus Promotes Cell Survival and Cellular Transformation by Suppressing Glycolysis

**DOI:** 10.1371/journal.ppat.1005648

**Published:** 2016-05-17

**Authors:** Ying Zhu, Suzane Ramos da Silva, Meilan He, Qiming Liang, Chun Lu, Pinghui Feng, Jae U. Jung, Shou-Jiang Gao

**Affiliations:** 1 Department of Molecular Microbiology and Immunology, Keck School of Medicine, University of Southern California, Los Angeles, California, United States of America; 2 Department of Microbiology and Immunology, Nanjing Medical University, Nanjing, Jiansu, People's Republic of China; Wistar Institute, UNITED STATES

## Abstract

Aerobic glycolysis is essential for supporting the fast growth of a variety of cancers. However, its role in the survival of cancer cells under stress conditions is unclear. We have previously reported an efficient model of gammaherpesvirus Kaposi’s sarcoma-associated herpesvirus (KSHV)-induced cellular transformation of rat primary mesenchymal stem cells. KSHV-transformed cells efficiently induce tumors in nude mice with pathological features reminiscent of Kaposi’s sarcoma tumors. Here, we report that KSHV promotes cell survival and cellular transformation by suppressing aerobic glycolysis and oxidative phosphorylation under nutrient stress. Specifically, KSHV microRNAs and vFLIP suppress glycolysis by activating the NF-κB pathway to downregulate glucose transporters GLUT1 and GLUT3. While overexpression of the transporters rescues the glycolytic activity, it induces apoptosis and reduces colony formation efficiency in softagar under glucose deprivation. Mechanistically, GLUT1 and GLUT3 inhibit constitutive activation of the AKT and NF-κB pro-survival pathways. Strikingly, GLUT1 and GLUT3 are significantly downregulated in KSHV-infected cells in human KS tumors. Furthermore, we have detected reduced levels of aerobic glycolysis in several KSHV-infected primary effusion lymphoma cell lines compared to a Burkitt’s lymphoma cell line BJAB, and KSHV infection of BJAB cells reduced aerobic glycolysis. These results reveal a novel mechanism by which an oncogenic virus regulates a key metabolic pathway to adapt to stress in tumor microenvironment, and illustrate the importance of fine-tuning the metabolic pathways for sustaining the proliferation and survival of cancer cells, particularly under stress conditions.

## Author Summary

KSHV is causally associated with the development of Kaposi’s sarcoma and primary effusion lymphoma; however, the mechanism underlying KSHV-induced malignant transformation remains unclear. The recent development of an efficient KSHV-induced cellular transformation model of primary rat mesenchymal stem cells should facilitate the delineation of KSHV-induced oncogenesis. In this report, we have used this model to investigate the metabolic pathways mediating the proliferation and survival of KSHV-transformed cells. In contrast to most other cancers that depend on aerobic glycolysis for their fast growth, we demonstrate that KSHV suppresses aerobic glycolysis and oxidative phosphorylation in the transformed cells. Significantly, suppression of aerobic glycolysis enhances the survival of the KSHV-transformed cells under nutrient deprivation. Mechanistically, KSHV-encoded microRNAs and vFLIP suppress aerobic glycolysis by activating the NF-κB pathway to downregulate glucose transporters GLUT1 and GLUT3. We have further shown that GLUT1 and GLUT3 inhibit constitutive activation of the AKT and NF-κB pro-survival pathways. Strikingly, GLUT1 and GLUT3 are significantly downregulated in KSHV-infected cells in human KS tumors. Furthermore, we have detected reduced levels of aerobic glycolysis in several KSHV-infected primary effusion lymphoma cell lines and a KSHV-infected Burkitt’s lymphoma cell line BJAB. Our results reveal a novel mechanism by which an oncogenic virus regulates a key metabolic pathway to adapt to stress in tumor microenvironment, and illustrate the importance of fine-tuning the metabolic pathways for sustaining the proliferation and survival of cancer cells, particularly under nutrient stress microenvironment.

## Introduction

It has been recognized that metabolic reprogramming is a core hallmark of cancer[1]. The Warburg effect describes the dependence of cancer cells on aerobic glycolysis for their growth and proliferation[2]. Increased glucose uptake and aerobic glycolysis are widely observed in cancer and clinically exploited for diagnosis[3]. Aerobic glycolysis provides a fast supply of ATP to support the rapid growth and proliferation of cancer cells[3]. Recent works have shown that besides energy, cancer cells have special needs for macromolecular building blocks and maintenance of redox balance[4, 5]. Accordingly, metabolic adaptation in cancer cells has been extended beyond the Warburg effect[5]. Several types of cancers depend on glutamine or one carbon amino acids for growth and proliferation[4, 5].

Cancer cells often encounter a variety of stress conditions including low nutrients, low oxygen and excess byproducts in the microenvironment[4, 6]. To optimize the growth, proliferation and survival under diverse conditions, cancer cells must fine-tune the metabolic pathways. Hyperactivation of metabolic pathways can generate toxic products that are detrimental to the cancer cells[6]. For examples, overflow of oxidative phosphorylation produces reactive oxidative species while excess of aerobic glycolysis leads to the buildup of lactate and low pH in the microenvironment[6]. How cancer cells regulate metabolic pathways to adapt to different stress conditions is not entirely clear.

Kaposi’s sarcoma-associated herpesvirus (KSHV) is an oncogenic virus associated with several cancers including Kaposi’s sarcoma (KS) and primary effusion lymphoma (PEL)[7]. Infection by KSHV has become an excellent model for understanding the mechanism of oncogenesis. Experimentally, KSHV can efficiently infect and transform primary rat mesenchymal precursor cells (MM) and human mesenchymal stem cells[8, 9]. KSHV-transformed MM cells (KMM) efficiently induce tumors with features closely resembling KS[8]. In KS tumors, PEL and KMM tumors, most of tumor cells are latently infected by KSHV. These cells have restricted expression of viral genes including vFLIP (ORF71), vCyclin (ORF72), LANA (ORF73) and 12 precursor microRNAs (pre-miRNAs)[8, 10, 11]. Genetic analyses have revealed that viral miRNAs and vCyclin are critical for KSHV-induced oncogenesis by regulating cell cycle and apoptosis[10], and overriding cell contact inhibition[12], respectively.

KSHV infection induces Warburg effect in human endothelial cells (ECs) and lipogenesis in ECs and PEL cells, and these altered metabolic processes are required for maintaining KSHV latency[13–15]. Among the KSHV-encoded products, the miRNA cluster decreases mitochondria biogenesis and induces aerobic glycolysis in ECs[16]. KSHV also induces glutamate secretion in ECs[17]. Nevertheless, in these studies, KSHV infection did not lead to cellular transformation. Thus, whether metabolic reprogramming is essential for KSHV-induced cellular transformation remains unknown.

In this study, we have discovered that KSHV suppresses aerobic glycolysis and oxidative phosphorylation in KSHV-transformed cells and this reprogramed metabolic pathway is essential for adaptation to glucose deprivation. These findings indicate that fine-tuning of metabolic pathways is essential for the proliferation and survival of cancer cells, particularly under stress conditions.

## Results

### KSHV-Transformed Cells Have Reduced Levels of Glycolysis and Oxidative Phosphorylation, and Do Not Require Glucose for Proliferation and Formation of Colonies in Softagar

**Fig 1:**
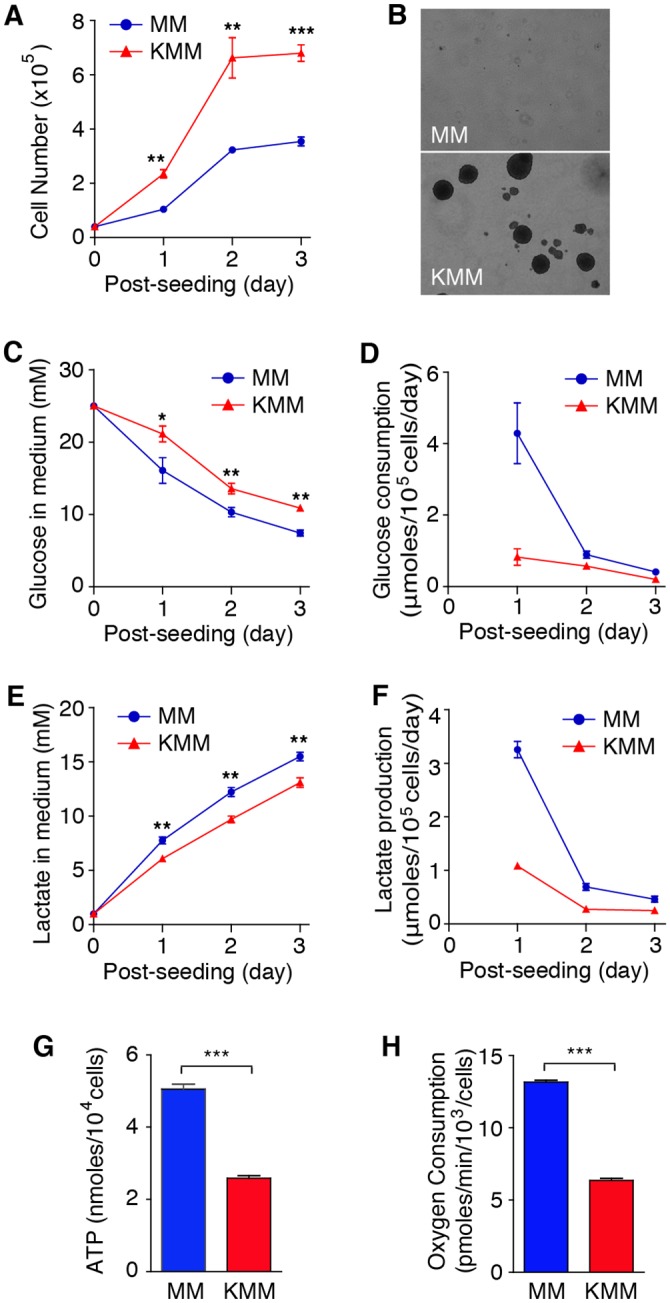
KSHV-transformed cells have reduced levels of glucose consumption, lactate production, oxygen consumption and intracellular ATP. (**A**). KMM cells have faster proliferation rate than MM cells. Cells were seeded at 4×10^4^ cells/well in 24-well plates and counted daily without medium changes. (**B**). KMM but not MM cells form colonies in softagar. MM and KMM cells at 2x10^4^ cells/well were plated in softagar in 6 well-plates for 14 days. Representative pictures captured at 40x magnification are presented in the left panel. (**C-F**). KMM cells have reduced levels of glucose consumption (**C** and **D**) and lactate production (**E** and **F**) measured by enzymatic assays in the medium (**C** and **E**) or adjusted for cell number (**D** and **F**). (**G-H**). KMM cells have reduced levels of intracellular ATP and oxygen consumption. Levels of intracellular ATP (**G**) and oxygen consumption (**H**) of MM and KMM were determined at day 2 post seeding. All data are presented as mean ± s.e.m. from three (n = 3, **A**, and **C-G**) or four (n = 4, **H**) independent experiments, each with three repeats. Representative images from three independent experiments with similar results are presented (**B**). * *P* < 0.05; ** *P* < 0.01; *** *P* < 0.001.

KSHV-transformed cells (KMM) proliferated significantly faster than their uninfected/untransformed counterparts (MM), and KMM but not MM cells lost contact-inhibition and formed colonies in softagar (Fig 1A and 1B)[8]. To determine the metabolic state of KSHV-transformed cells, we examined the consumption of glucose, the main carbon source for most normal and cancer cells. In normal cells, glucose flows through glycolysis and tricarboxylic acid (TCA) cycle to generate ATP and NADH with the latter further serving as a substrate for oxidative phosphorylation to produce additional ATP, a process that consumes oxygen[4]. However, many cancer cells have fast ATP production through a higher rate of aerobic glycolysis, resulting in higher rates of glucose consumption and lactate production despite the presence of oxygen[3]. To our surprise, KMM cells consumed significantly less glucose than MM cells did (Fig 1C). This effect was even more dramatic after taking into account of cell proliferation rate (Fig 1D). KMM cells also produced less lactate, and had lower levels of intracellular ATP and oxygen consumption compared to MM cells (Fig 1E–1H). Thus, despite their rapid proliferation, KMM cells consume less glucose, and have lower activities of aerobic glycolysis and oxidative phosphorylation.

**Fig 2:**
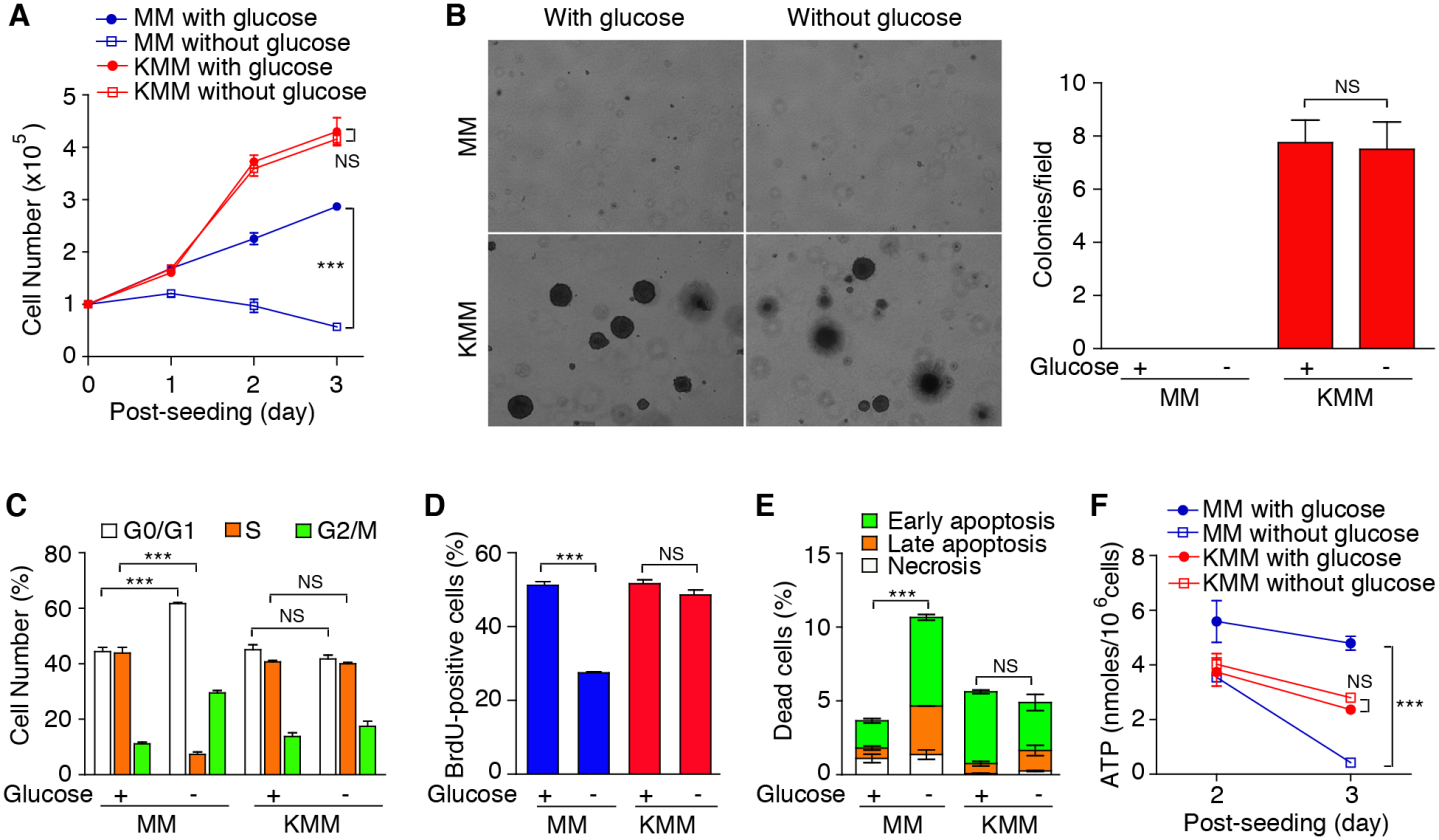
KMM cells do not require glucose for proliferation, survival and formation of colonies in Softagar. (**A**). KMM cells do not require glucose for proliferation. MM and KMM cells seeded at 10^5^ cells/well in 6-well plates in complete media (25 mM glucose) which were replaced with glucose-free or complete medium the following day, and cell numbers were counted daily. (**B**). KMM cells do not require glucose for formation of colonies in softagar. MM and KMM cells were plated in softagar in the presence or absence of glucose as described in Fig 1B. Representative pictures captured at 40x magnification are presented in the left panel. Colonies with diameter >50 μm were counted and colony numbers in each field are presented in the right panel. (**C-D**). MM but not KMM cells are cell cycle arrested following glucose deprivation. Cell cycle distribution and BrdU incorporation were analyzed by flow cytometry following 24 h glucose deprivation. (**E**). Glucose deprivation induces apoptosis in MM but not KMM cells. Apoptotic cells were detected by Annexin V staining following 48 h of glucose deprivation. (**F**). Intracellular ATP levels decrease in MM but not KMM cells following glucose deprivation. Intracellular ATP levels were determined in cells grown in media with or without glucose at day 2 and 3. All data are presented as mean ± s.e.m. from three (n = 3) independent experiments, each with three repeats. NS, not significant; *** *P* < 0.001.

Because KMM cells consumed less glucose, we postulated that they might not require glucose to support their proliferation. Indeed, glucose deprivation affected neither the proliferation nor colony formation of KMM cells in softagar while it caused proliferation arrest of MM cells (Fig 2A and 2B). Glucose deprivation caused G1 arrest, reduced BrdU incorporation, increased apoptotic cells and decreased the intracellular ATP level in MM but not KMM cells (Fig 2C–2F). Collectively, these results indicate that KSHV has reprogramed the cellular metabolic pathways following cellular transformation.

### KSHV miRNAs and vFLIP Mediate Inhibition of Aerobic Glycolysis and Oxidative Phosphorylation, as well as Glucose-Independent Proliferation and Cellular Transformation

**Fig 3:**
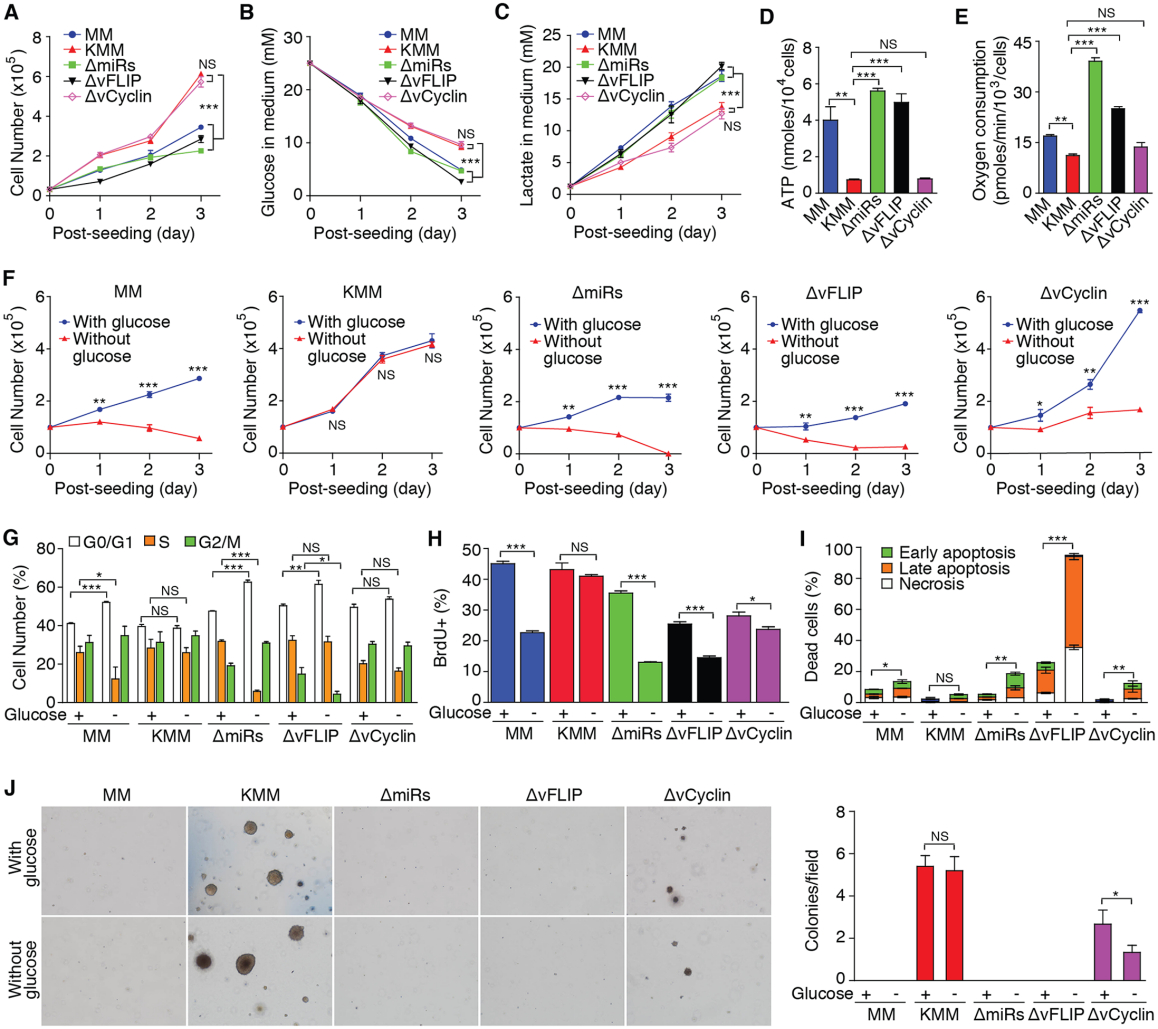
KSHV miRNAs and vFLIP mediate inhibition of glucose consumption, aerobic glycolysis and oxidative phosphorylation, as well as glucose-independent cell proliferation and cellular transformation. (**A**). Cell proliferation of MM cells and those infected by recombinant KSHV including wild-type (KMM), and mutants with a deletion of a cluster of 10 precursor miRNAs (ΔmiRs), vFLIP (ΔvFLIP) or vCyclin (ΔvCyclin) under normal culture conditions as described in Fig 1A. (**B-E**). Levels of glucose consumption (**B**), lactate production (**C**), intracellular ATP (**D**) and oxygen consumption (**E**) of cells infected by different recombinant viruses determined as described in Fig 1C–1H. (**F**). Cell proliferation of cells infected by different recombinant viruses in the presence or absence of glucose measured as described in Fig 2A. (**G-I**). Cell cycle profiles (**G**), BrdU incorporation (**H**) and apoptosis (**I**) of cells infected by different recombinant viruses measured as described in Fig 2C–2E. (**J**). Formation of colonies in softagar of cells infected by different recombinant viruses in the presence or absence of glucose measured as described in Fig 2B. All data are presented as mean ± s.e.m. from three (n = 3, **A-D**, and **F-J**) or four (n = 4, **E**) independent experiments, each with three repeats. Representative images from three independent experiments with similar results are presented (**J**). NS, not significant; * *P* < 0.05; ** *P* < 0.01; *** *P* < 0.001.

KMM cells are latently infected by KSHV and mostly express only viral latent genes/products including vFLIP, vCyclin, LANA and miRNAs[8]. To identify KSHV genes/products that mediate metabolic reprogramming, we generated MM cells latently infected by KSHV mutants containing individual deletion of vFLIP, vCyclin or 10 of the 12 pre-miRNAs (miR-K1-9 and 11)[10, 12, 18]. We were unable to obtain cells stably infected by a mutant of LANA because of its essential role in persistent infection[19, 20]. Under normal culture condition, deletion of vFLIP or the miRNA cluster reduced cell proliferation rates to levels similar to those of MM cells (Fig 3A). Deletion of vCyclin had no effect on cell proliferation though a slower rate was observed at contact-inhibited high cell density[12]. We further examined the metabolic states of these cells. Deletion of vFLIP or the miRNA cluster but not vCyclin increased glucose consumption, lactate production, intracellular ATP and oxygen consumption to levels close to or even higher than those of MM cells (Fig 3B–3E). Furthermore, deletion of vFLIP or the miRNA cluster sensitized the cells to glucose deprivation, causing cell proliferation arrest similar to MM cells (Fig 3F). While vCyclin mutant cells continued to proliferate upon glucose deprivation, they did so at a rate slower than that of KMM cells (Fig 3F). Consistently, glucose deprivation caused G1 arrest, reduced BrdU incorporation and increased apoptotic cells in cells of vFLIP and miRNA cluster mutants (Fig 3G–3I). Interestingly, the basal level of dead cells in the vFLIP mutant cells were higher than those of MM and KMM cells (25% *vs* 8% and 3%, respectively), and were further increased upon glucose deprivation, reaching as high as 95% (Fig 3I), which could be attributed to the oncogenic stress in KSHV-transformed cells[10] and vFLIP activation of the NF-κB[21, 22]. In contrast, glucose deprivation had minimal effect on cell cycle progression and BrdU incorporation of vCyclin mutant cells (Fig 3G and 3H); however, it increased apoptotic cells to a level similar to that of MM cells (Fig 3I), which might explain the slower proliferation rate of vCyclin mutant cells than KMM cells (Fig 3F). We further correlated the metabolic states of these cells with cellular transformation. Cells of both vFLIP and miRNA cluster mutants failed to form any colonies in softagar, a phenotype resembling that of MM cells (Fig 3J). vCyclin mutant cells formed significantly less and smaller colonies than KMM cells did in normal culture condition as previously reported[12] but continued to form colonies upon glucose deprivation albeit at a reduced efficiency (Fig 3J). Together, these results indicate that both vFLIP and the miRNA cluster mediate KSHV reprograming of metabolic pathways, contributing to KSHV-induced glucose-independent cell proliferation, survival and cellular transformation. While vCyclin can override contact inhibition to promote cellular transformation[12], it does not contribute to KSHV reprogramming of metabolic pathways.

### vFLIP and the miRNA Cluster Mediate KSHV Downregulation of GLUT1 and GLUT3 in a NF-κB-Dependent Manner

**Fig 4:**
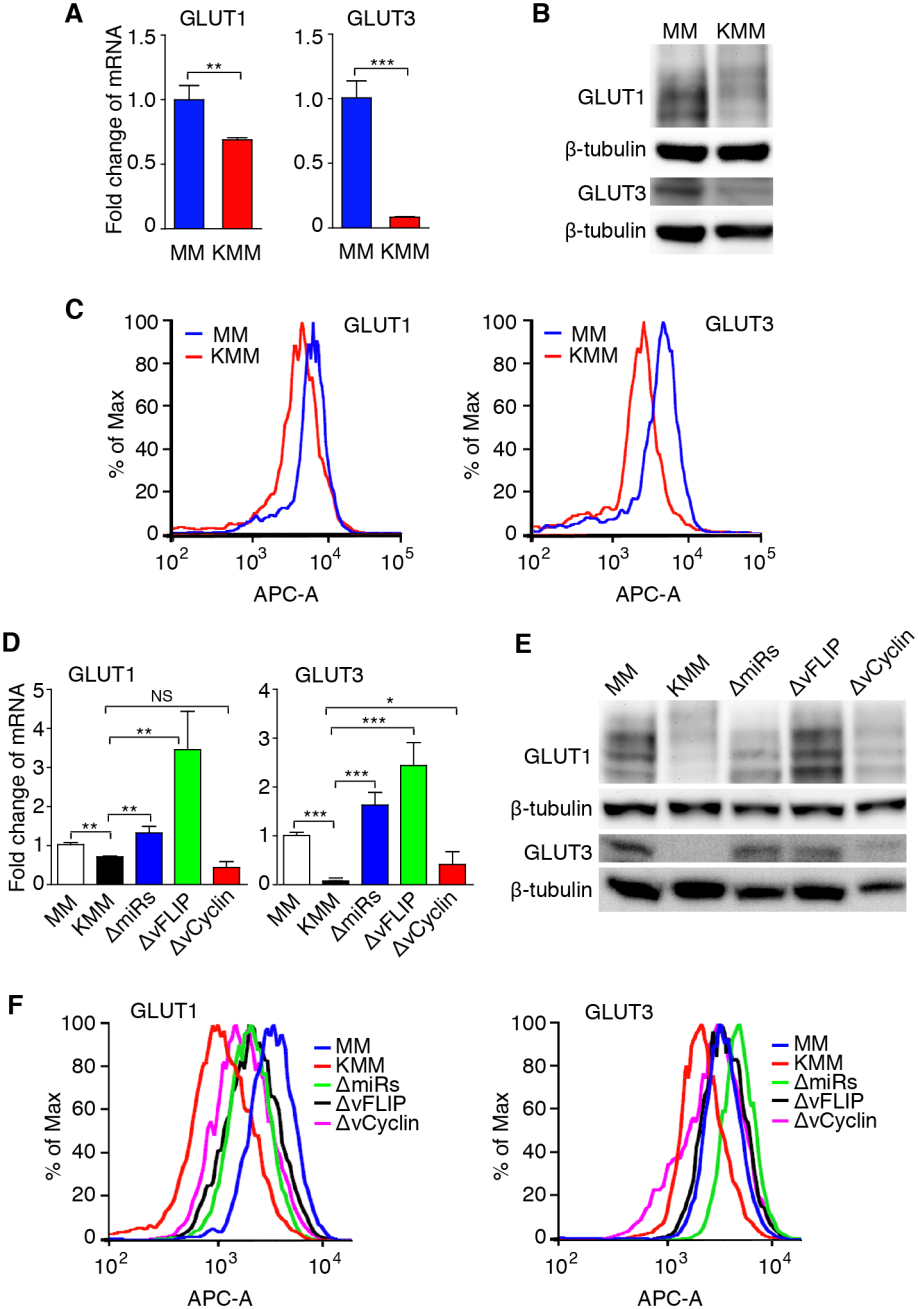
KSHV vFLIP and the miRNA cluster mediate KSHV downregulation of GLUT1 and GLUT3. (**A-C**). Analysis of GLUT1 and GLUT3 expression in MM and KMM cells by RT-qPCR (**A**), Western-blot (**B**) and flow cytometry (**C**). (**D-F**). Analysis of GLUT1 and GLUT3 expression in cells infected by different recombinant viruses by RT-qPCR (**D**), Western-blot (**E**) and flow cytometry (**F**). β-actin and β-tubulin were used as internal controls for RT-qPCR and Western-blot, respectively. For panels **C** and **F**, the Y-axis is shown as normalized cell numbers. All data are presented as mean ± s.e.m. from three (n = 3, **A** and **D**) independent experiments, each with three repeats. Representative images from three independent experiments with similar results are presented (**B, C, E,** and **F**). NS, not significant; * *P* < 0.05; ** *P* < 0.01; *** *P* < 0.001.

To identify the mechanism of KSHV inhibition of aerobic glycolysis and oxidative phosphorylation, we examined changes of gene expression of key enzymes in the glycolysis pathway following KSHV transformation. All glycolysis enzymes either had minimal change or were upregulated (S1 Fig); hence, they were unlikely the candidates that mediated KSHV suppression of glycolysis. GLUT1 and GLUT3 directly mediate glucose uptake, which is the first step in the glycolysis pathway[4]. Downregulation of GLUT1 and GLUT3 was observed at mRNA and protein levels (Fig 4A–4C). Importantly, deletion of vFLIP or the miRNA cluster was sufficient to restore the GLUT1 and GLUT3 expression levels (Fig 4D and 4E). Interestingly, the mRNA levels of both GLUT1 and GLUT3 detected by reverse transcription quantitative real time PCR (RT-qPCR) and the protein level of GLUT1 detected by Western-blot were even higher in vFLIP mutant cells than in MM cells. The results of flow cytometry were inconsistent, which were probably due to the fact that the antibodies detected surface expression while RT-qPCR and Western-blot detected the total levels of mRNA and protein in cells, respectively. Deletion of vCyclin did not affect GLUT1 and GLUT3 mRNA expression levels but marginally increased their protein levels (Fig 4D–4F). These results indicate that vFLIP and the miRNA cluster mediate KSHV downregulation of GLUT1 and GLUT3.

**Fig 5:**
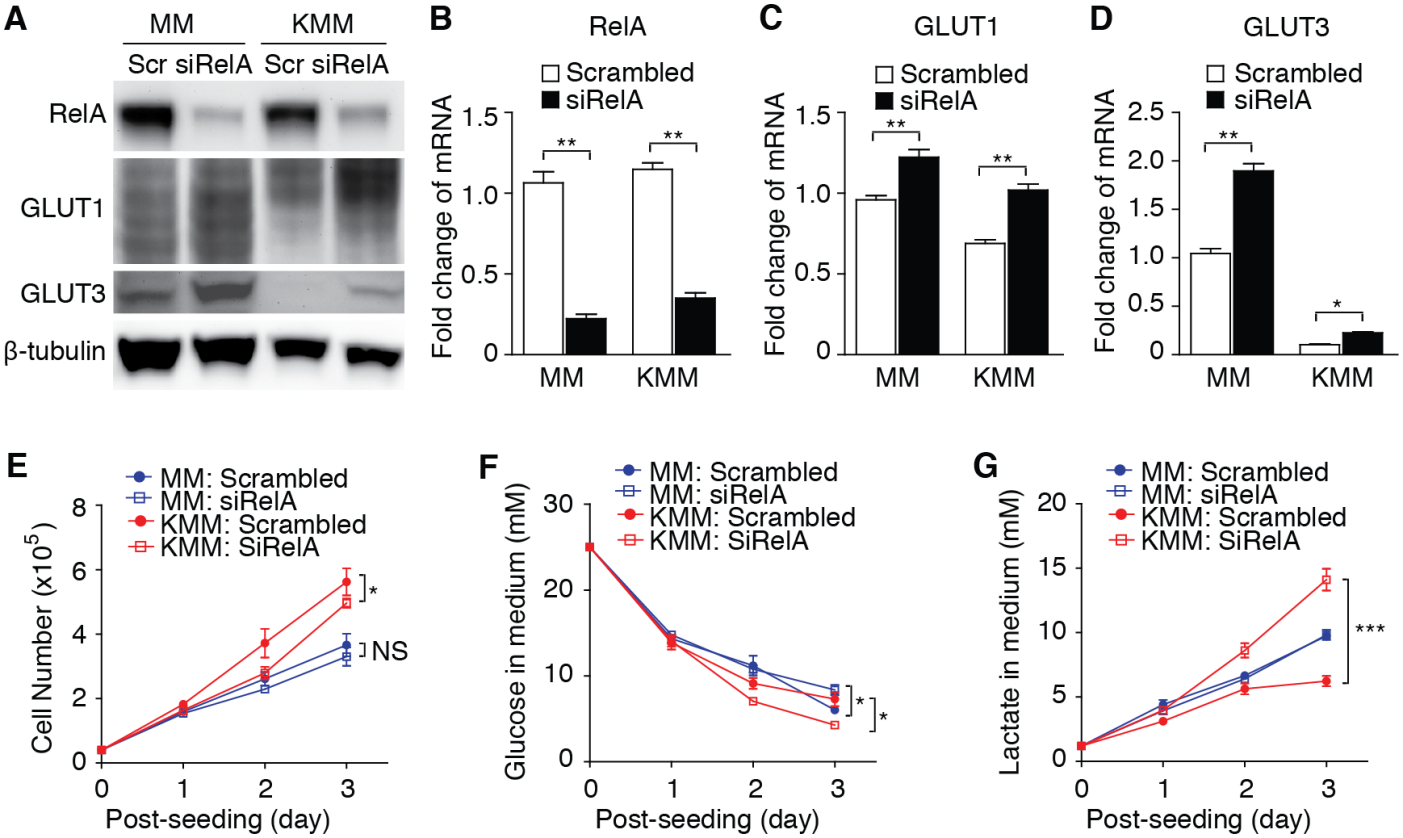
Knock down of RelA increases the expression of GLUT1 and GLUT3, and aerobic glycolysis. (**A**). Analysis of RelA, GLUT1 and GLUT3 proteins in MM and KMM cells by Western-blot following knock down of RelA. Detection of RelA, GLUT1 and GLUT3 proteins in MM and KMM cells following transfection with a siRNA to RelA (siRelA) or a scrambled control (Scr) for 3 days. β-tubulin was used as an internal control for loading. (**B-C**). Analysis of RelA (**B**), GLUT1 (**C**) and GLUT3 (**D**) mRNAs in MM and KMM cells by RT-qPCR following knock down of RelA. Cells were treated as described in (**A**). β-actin was used as an internal control for qPCR. (**E**). Knock down of RelA decrease cell proliferation of KMM but not MM cells. Cell proliferation were examined following knock down of RelA. (**F-G**) Knock down of RelA increases glucose consumption (**F**) and lactate production (**G**) in KMM cells. Glucose consumption and lactate production were determined as described in Fig 1C and 1E following knock down of RelA. Experiments were repeated three times, each with three repeats and representative results were presented. * *P* < 0.05; ** *P* < 0.01; *** *P* < 0.001.

To investigate the mechanism of KSHV downregulation of GLUT1 and GLUT3, we searched for a common pathway regulated by vFLIP and the miRNA cluster. Both vFLIP and the miRNA cluster activate the NF-κB pathway[21–23], and both are required for the maximal activation of the NF-κB pathway in KSHV-transformed cells[10]. Because knock down of RelA, a key component of the NF-κB complexes, is sufficient to inhibit the NF-κB pathway in KMM cells[10], we examined the effect of knock down of RelA on the expression of GLUT1 and GLUT3 (Fig 5A and 5B). Knock down of RelA significantly increased the protein and mRNA expression levels of both GLUT1 and GLUT3 (Fig 5A–5D). As previously reported[10], knock down of RelA slightly decreased cell proliferation of KMM cells but had no effect on MM cells (Fig 5E). Importantly, knock down of RelA increased glucose consumption and lactate production in KMM cells (Fig 5F and 5G). These effects were even more obvious when adjusted for cell numbers. In MM cells, knock down of RelA slightly increased the glucose consumption but had no detectable effect on lactate production.

**Fig 6:**
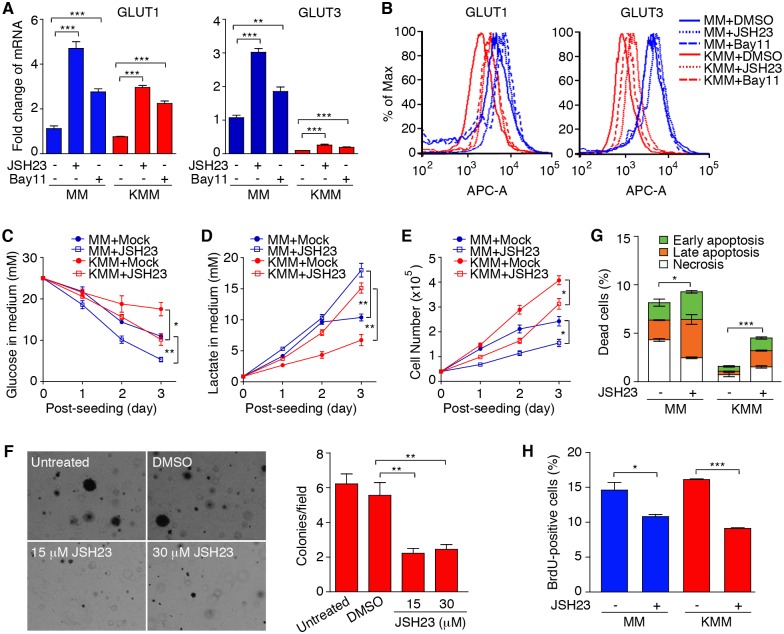
The NF-κB pathway mediates KSHV suppression of GLUT1 and GLUT3, and aerobic glycolysis. (**A-B**). Inhibition of the NF-κB pathway increases the expression of GLUT1 and GLUT3. MM and KMM cells were treated with NF-κB inhibitors JSH23 (30 μM) or BAY 11–7082 (2 μM), and examined for the expression of GLUT1 and GLUT3 expression by RT-qPCR at 8 h post-treatment (**A**) and flow cytometry at 24 h post-treatment (**B**). Y-axis in (**B**) is shown as normalized cell numbers. β-actin was used as internal controls for qPCR. (**C-D**). Inhibition of the NF-κB pathway increases glucose consumption (**C**) and lactate production (**D**). MM and KMM cells were treated with NF-κB inhibitor JSH23 (30 μM) and glucose consumption and lactate production were determined as described in Fig 1C and 1E. (**E-F**). Inhibition of the NF-κB pathway reduces cell proliferation and inhibits cellular transformation. Cell proliferation (**E**) and colony formation in softagar (**F**) were examined in the presence of JSH23 (30 μM) as described in Fig 1A and 1B. (**G-H**). Inhibition of the NF-κB pathway increases apoptosis and reduces BrdU incorporation. Apoptosis (**G**) and BrdU incorporation (**H**) of MM and KMM cells were examined following treatment of JSH23 (30 μM) for 48 h as described in Fig 2D and 2E. All data are presented as mean ± s.e.m. from three (n = 3, **A**, and **C-H**) independent experiments, each with three repeats. Representative images from three independent experiments with similar results are presented (**B** and **F**). * *P* < 0.05; ** *P* < 0.01; *** *P* < 0.001.

To confirm the above results, we carried out pharmacological inhibition of the NF-κB pathway with two specific inhibitors, JSH-23 and BAY11-7082. Both inhibitors significantly induced the expression of GLUT1 and GLUT3 at mRNA and protein levels (Fig 6A and 6B). Interestingly, neither knockdown of RelA nor the NF-κB inhibitors fully rescued the expression of GLUT3 in KMM, suggesting that another pathway, besides the NF-κB pathway, might be involved in the inhibition of GLUT3 expression in KMM cells. Inhibition of the NF-κB pathway increased glucose consumption and lactate production in both MM and KMM cells (Fig 6C and 6D). Importantly, the increased glucose consumption and lactate production rates were correlated with reduced cell proliferation rates in both MM and KMM cells and a reduced efficiency of colony formation of KMM cells in softagar (Fig 6E and 6F). Consistent with these results, inhibition of the NF-κB pathway sensitized KMM cells to apoptosis and inhibited BrdU incorporation (Fig 6G and 6H). Thus, the NF-κB pathway promotes cell proliferation and cellular transformation at least in part by inhibiting the expression of GLUT1 and GLUT3 to limit the glucose consumption in KMM cells.

### Overexpression of GLUT1 or GLUT3 Increases Glucose Consumption and Lactate Production, and Sensitizes KMM Cells to Apoptosis upon Glucose Deprivation

**Fig 7:**
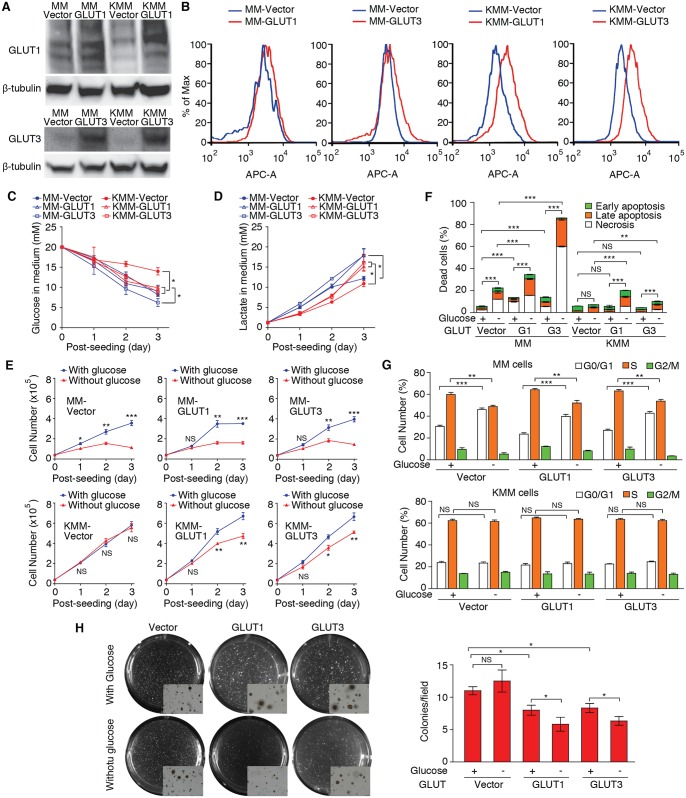
Overexpression of GLUT1 or GLUT3 enhances aerobic glycolysis, and sensitizes KMM cells to apoptosis upon glucose deprivation. (**A-B**). Stable expression of GLUT1 and GLUT3 in MM and KMM cells. Cells with stable expression of GLUT1 or GLUT3 were analyzed by Western-blot (**A**) and flow cytometry (**B**). Y-axis in (**B**) is shown as normalized cell numbers. β-tubulin was used as internal controls for Western-blot. (**C-D**). Overexpression of GLUT1 or GLUT3 increases glucose consumption (**C**) and lactate production (**D**). MM and KMM cells with stable expression of GLUT1 or GLUT3 were examined for glucose consumption and lactate production as described in Fig 1C and 1E. (**E-G**). Overexpression of GLUT1 or GLUT3 sensitizes KMM cells to glucose deprivation shown in cell proliferation (**E**) and apoptosis (**F**) but not cell cycle distribution (**G**). Cell proliferation, apoptosis and cell cycle progression of MM and KMM cells stably expressing GLUT1 or GLUT3 were examined in the presence or absence of glucose as described in Fig 2A, 2C and 2E. (**H**). Overexpression of GLUT1 or GLUT3 reduces the efficiency of colony formation of KMM cells in softagar. Colony formation of KMM cells was examined in the presence or absence of glucose as described in Fig 1A. All data are presented as mean ± s.e.m. from three (n = 3, **C-H**) independent experiments, each with three repeats. Representative images from three independent experiments with similar results are presented (**A, B,** and **H**). NS, not significant; * *P* < 0.05; ** *P* < 0.01; *** *P* < 0.001.

To confirm if downregulation of GLUT1 and GLUT3 mediated KSHV inhibition of glucose consumption and lactate production, we overexpressed GLUT1 and GLUT3 in MM and KMM cells (Fig 7A and 7B). Overexpression of GLUT1 or GLUT3 was sufficient to increase glucose consumption and lactate production in KMM cells but the results were inconsistent with MM cells, which might reflect their cell surface expression levels (Fig 7C and 7D). While overexpression of GLUT1 or GLUT3 neither significantly affected cell proliferation of both MM and KMM cells under normal culture condition nor altered the sensitivity of MM cells to glucose deprivation, it reduced cell proliferation of KMM cells upon glucose deprivation (Fig 7E). Consistently, glucose deprivation increased the number of apoptotic cells in KMM cells with overexpression of GLUT1 or GLUT3 (Fig 7F). As expected, MM cells were sensitive to glucose deprivation. Overexpression of GLUT1 or GLUT3 increased the basal number of apoptotic cells in MM cells, which was further increased upon glucose deprivation (Fig 7F). Interestingly, overexpression of GLUT1 or GLUT3 had no effect on cell cycle progression in KMM cells in either normal culture condition or in medium deprived of glucose (Fig 7G). Thus, the glucose transporters regulate cell survival rather than cell cycle progression under nutritional stress conditions in KSHV-transformed cells. Finally, overexpression of GLUT1 or GLUT3 in KMM cells slightly increased the sizes of some colonies but significantly reduced the number of colonies in softagar in normal culture medium, which was further reduced upon glucose deprivation (Fig 7H). Taken together, these results indicate that suppression of GLUT1 and GLUT3 expression confers KMM cells lower levels of glucose consumption and lactate production, and endow them the potential for glucose-independent cell proliferation, survival and cellular transformation.

### Overexpression of GLUT1 or GLUT3 Impairs the AKT-NF-κB Survival Pathway

**Fig 8:**
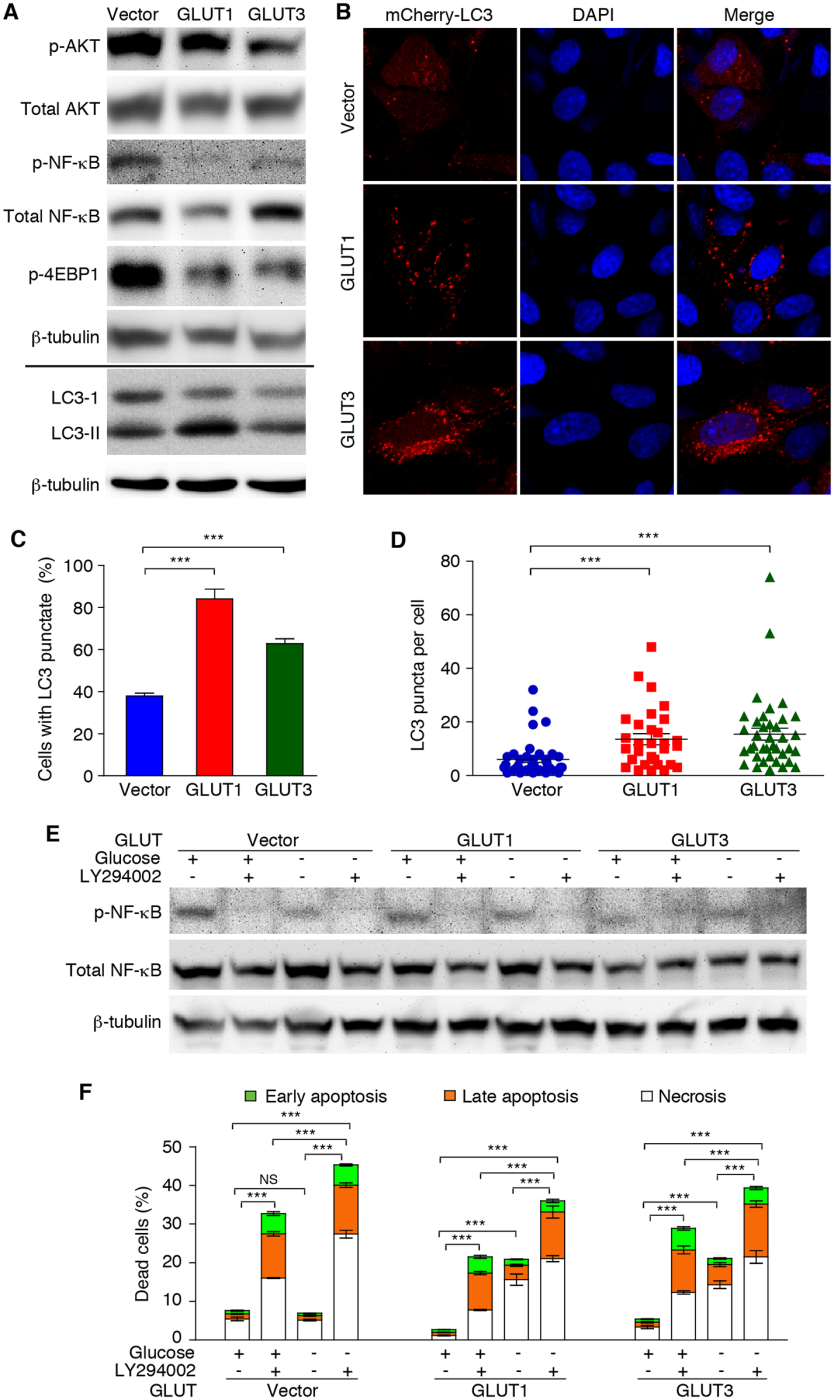
Overexpression of GLUT1 or GLUT3 impairs the AKT-NF-κB pro-survival pathway. (**A**). Effect of GLUT1 and GLUT3 on the AKT-NF-κB pro-survival pathway. Cell lysates from KMM-vector, KMM-GLUT1 and KMM-GLUT3 cells cultured under normal conditions were analyzed by Western-blot with the specified antibodies. β-tubulin was used as internal controls. (**B-D**). Overexpression of GLUT1 or GLUT3 increases autophagy in KMM cells. KMM-vector, KMM-GLUT1 and KMM-GLUT3 cells transduced with mCherry-LC3 for 48 h were examined for the formation of LC3 punctate. Representative images were captured at 1,000x magnification using laser-scanning confocal microscopy (**B**). The percentage of cells with LC3 punctate (**C**) and the number of LC3-positive dots per cell (**D**) were counted. (**E**). Glucose deprivation and inhibition of PI3K reduce NF-κB activation. Total and phosphorylation of NF-κB in KMM-vector, KMM-GLUT1 and KMM-GLUT3 cells in the presence or absence of glucose as well as with and without treatment with 12.5 μM PI3K inhibitor LY294002 were examined by Western-blot at 48 h post-treatment. (**F**). Glucose deprivation and inhibition of PI3K sensitize KMM cells to apoptosis. KMM-vector, KMM-GLUT1 and KMM-GLUT3 cells in the presence or absence of glucose as well as with and without treatment with 12.5 μM PI3K inhibitor LY294002 were examined for apoptosis at 72 h post-treatment. All data are presented as mean ± s.e.m. from three (n = 3, **C, D,** and **F**) independent experiments, each with three repeats. Representative images from three independent experiments with similar results are presented (**A, B,** and **E**). NS, not significant; *** *P* < 0.001.

To determine the mechanism mediating GLUT1 and GLUT3 regulation of the survival of KMM cells, we examined two main cell survival pathways AKT and NF-κB. Overexpression of GLUT1 or GLUT3 in KMM cells reduced the phospho-AKT level (Fig 8A). The AKT downstream targets phospho-NF-κB p65 and phospho-4EBP1 were also reduced (Fig 8A). Accordingly, we observed increased levels of autophagy, which is regulated by the AKT pathway, in these cells. Specifically, there were increased LC3-II/LC3-I ratio, more cells with the typical LC3 punctate staining and increased number of punctates per cell in KMM cells with overexpression of GLUT1 or GLUT3 (Fig 8A–8D). These results indicate that GLUT1 and GLUT3 impair the AKT and NF-κB survival pathways in KMM cells. To determine if AKT pathway mediated the activation of NF-κB pathway in KMM cells, we treated cells with an inhibitor of the AKT upstream activator PI3K. Interestingly, the PI3K inhibitor and glucose deprivation reduced the total and phosphorylated p65 levels (Fig 8E). The PI3K inhibitor and glucose deprivation synergized with each other to further reduce the total and phosphorylated p65 levels. As shown in Fig 7F, KMM cells with overexpression of GLUT1 or GLUT3 were sensitive to glucose deprivation with increased numbers of apoptotic cells (Fig 8F). Treatment with the PI3K inhibitor alone was sufficient to increase the numbers of apoptotic cells in these cells, and further sensitized them to glucose deprivation. While KMM cells were resistant to glucose deprivation (Fig 2E), treatment with the PI3K inhibitor alone was sufficient to increase the number of apoptotic cells in KMM cells overexpressing the vector control, or GLUT1 and GLUT3, and further sensitized them to apoptosis upon glucose deprivation (Fig 8F). Collectively, these results indicate that the resistance of KMM cells to glucose deprivation is likely due to their reduced GLUT1 and GLUT3 levels, resulting in the enhanced persistent activation of the AKT-NF-κB pathway.

### GLUT1 and GLUT3 Are Downregulated in KSHV-Infected Tumor Cells in Human KS Tumors, and Aerobic Glycolysis Is Suppressed in KSHV-Infected PEL Cells

**Fig 9:**
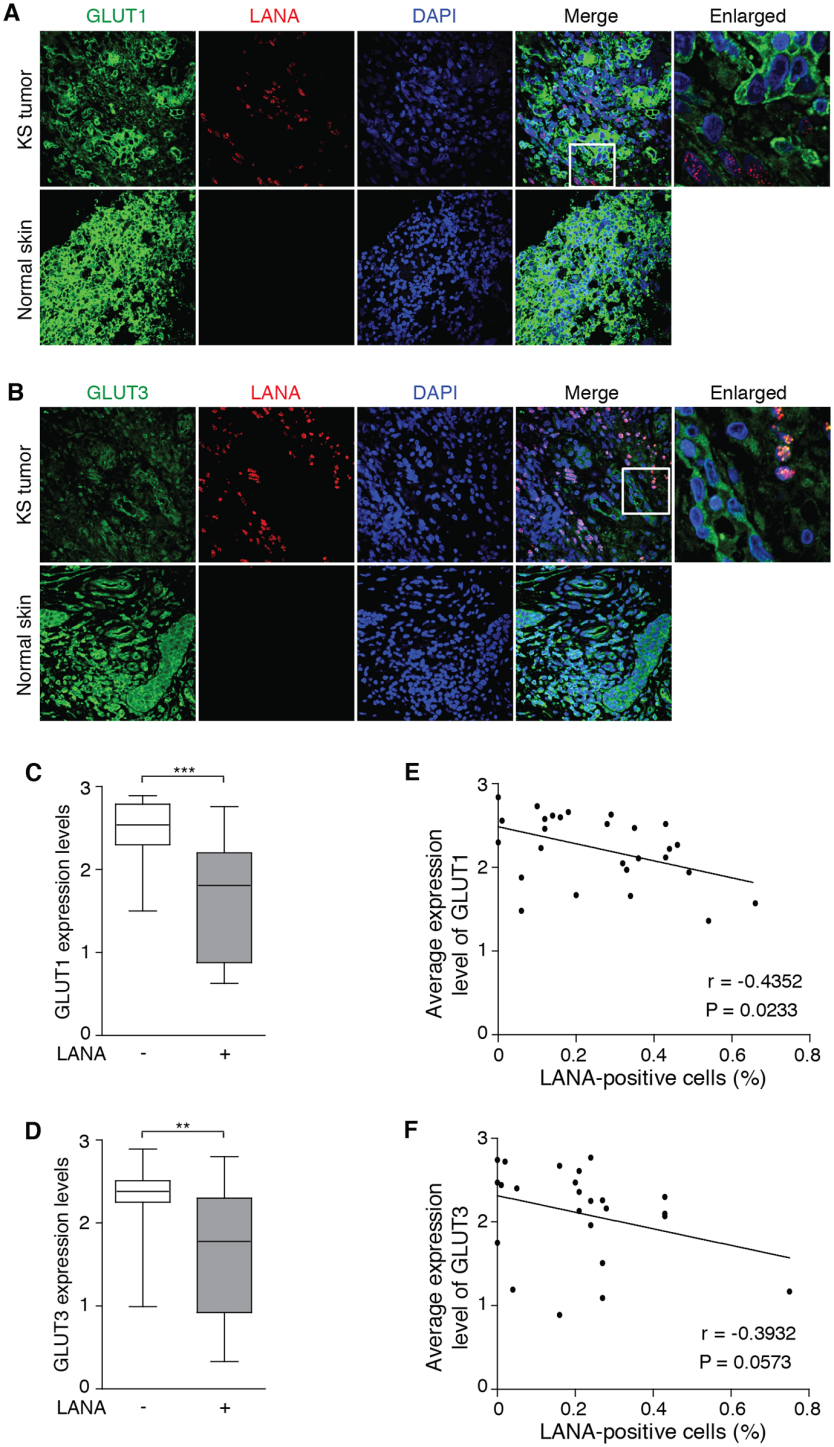
GLUT1 and GLUT3 are downregulated in KSHV-infected cells in human KS tumors. (**A-B**). Representative illustration of dual immunofluorescence detection of LANA and GLUT1 (**A**) or GLUT3 (**B**) in a normal human skin section and a KS tumor section. The tissue sections were counterstained with DAPI. Magnifications 600x. Boxed areas are enlarged. (**C**) Analysis of GLUT1 expression in LANA negative (-) and LANA positive (+) cells in KS tumors (n = 25). (**D**) Analysis of GLUT3 expression in LANA negative (-) and LANA positive (+) cells in KS tissues (n = 17). (**E-F**) Negative correlation of the average percentage of LANA-positive cells with the average expression level of GLUT1 (**E**) and GLUT3 (**F**) in KS tumors. For (**C** and **D**), the boxes represent the interquartile range (25-75^th^ centiles. The horizontal line inside the box indicates the median. The vertical whiskers extend to the maximum and minimum values. Statistical analysis was performed by Wilcoxon matched-pairs signed-ranks test. Expression levels of GLUT1 and GLUT3 were quantified based on immunofluorescence staining, using a modified His-score as described in the Materials and Methods. **P < 0.01; ***P < 0.001.

We have so far demonstrated that KSHV promotes cell survival under nutrient deprivation by downregulating GLUT1 and GLUT3 to suppress aerobic glycolysis. To demonstrate the pathological relevance of these observations, we examined the expression of GLUT1 and GLUT3 proteins in human KS tumors on a tissue array by dual-color immunofluorescence staining (Fig 9A and 9B). The expression of GLUT1 and GLUT3 was evaluated using a modified Histo-score (H-score) as described in the Materials and Methods. We observed significant downregulation of GLUT1 and GLUT3 in LANA-positive cells compared to LANA-negative cells in the KS tumors as well as adjacent uninvolved tissues (Fig 9C and 9D). A total of 27 specimens were retained following GLUT1 staining (S2 Fig). Of the 22 specimens that had robust LANA signal (detection of > 10 LANA-positive cells), 20 (90%) had significantly downregulation of GLUT1 in the LANA-positive cells compared to LANA-negative cells. Three specimens had weak LANA signal (detection of < 10 LANA-positive cells). Of the 2 specimens that had no detectable LANA signal (normal skin tissues), we detected strong GLUT1 signal. Among the specimens that had LANA-positive cells, the average GLUT1 signal was already negatively correlated with the numbers of LANA-positive cells (r = -0.5351, P = 0.0233 in Fig 9E). A total of 22 specimens were retained following GLUT3 staining (S3 Fig). Of the 17 specimens that had strong LANA signal (detection of >10 LANA-positive cells), 13 (76%) had significantly downregulation of GLUT3 in the LANA-positive cells compared to LANA-negative cells. Two specimens had weak LANA signal (detection of 10 or < 10 LANA-positive cells). Of the 3 specimens that had no detectable LANA signal (normal skin tissues), we detected strong GLUT3 signal. Among the specimens that had LANA-positive cells, there was already a tend of negative correlation between the average GLUT3 signal with the numbers of LANA-positive cells albeit it had not reached statistical significance (r = -0.3932, P = 0.0573 in Fig 9F). Together, these results suggest that KSHV suppression of aerobic glycolysis is present in the KS tumors.

**Fig 10:**
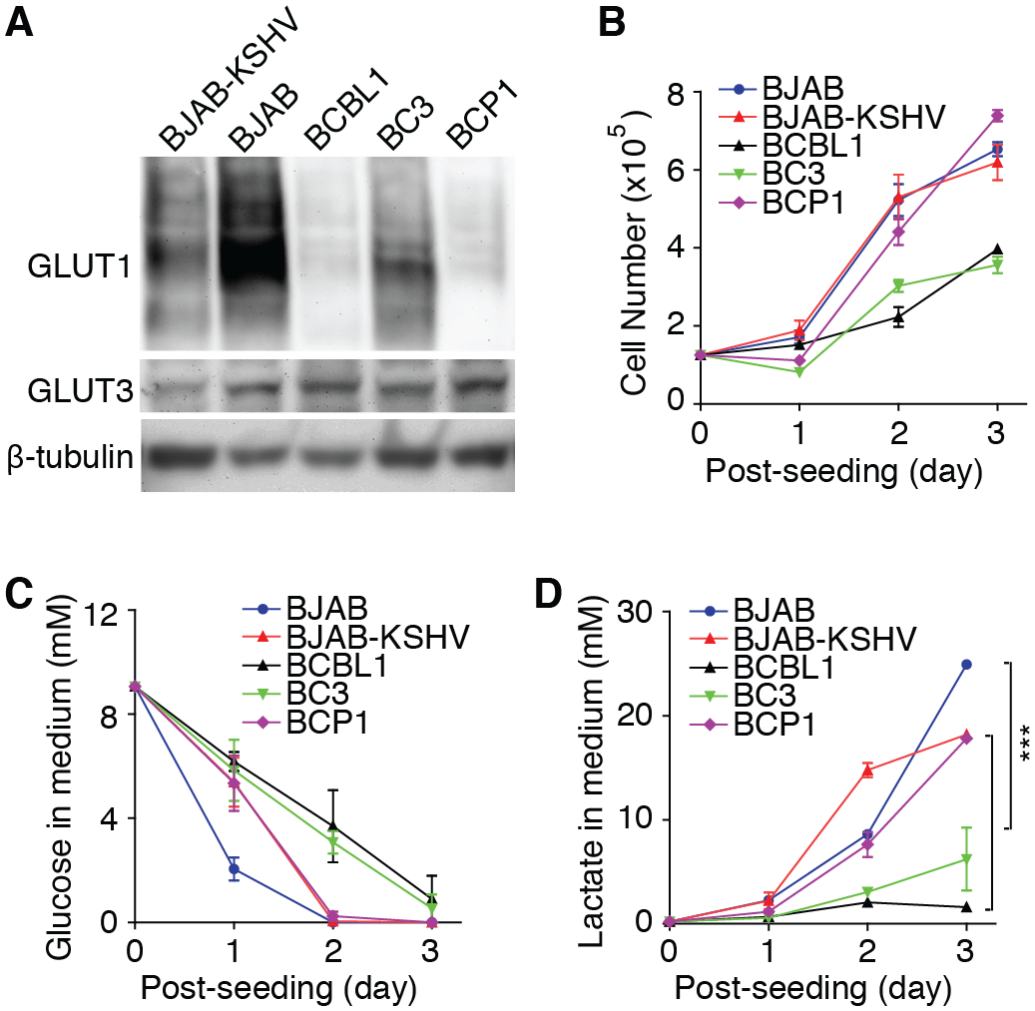
Suppression of aerobic glycolysis in PEL cells. (**A**). Downregulation of GLUT1 in PEL cell lines. Western-blot detection of GLUT1 and GLUT3 protein levels in PEL cell lines BCBL1, BC3 and BCP1, and uninfected and KSHV-infected BJAB cells. (**B**). Cell proliferation rates of PEL cell lines BCBL1, BC3 and BCP1, and uninfected and KSHV-infected BJAB cells. (**C-D**). KSHV-infected cells have reduced levels of glucose consumption (**C**) and lactate production (**D**). Glucose consumption and lactate production were determined as described in Fig 1C and 1E. Experiments were repeated three times, each with three repeats and representative results were presented. *** *P* < 0.001.

PEL is another malignancy associated with KSHV infection. Since primary PEL specimens are rare, we examined the expression of GLUT1 and GLUT3 in three PEL lines that are only infected by KSHV including BCBL1, BC3 and BCP1 cells (Fig 10A). Compared to BJAB, a KSHV-negative and EBV-negative Burkitt's lymphoma cell line, the expression of GLUT1 was downregulated in all PEL lines. However, the expression of GLUT3 had no obvious difference among the cell lines examined. As there is no appropriate control for the PEL cell lines, we examined BJAB cells infected by KSHV (BJAB-KSHV). KSHV infection downregulated the expression of both GLUT1 and GLUT3 in BJAB cells. BCBL1 and BC3 cells had slightly slower proliferation rates compared to other cell lines. However, by day 1 post-seeding, we observed slower glucose consumption rates in all KSHV-infected lines compared to BJAB (Fig 10C). By day 2 post-seeding, BJAB and BCP1 cells no longer had detectable glucose in the culture medium. We detected a higher level of lactate production by BJAB cells than those of all the KSHV-infected cell lines at day 3 post-seeding (Fig 10D). These results indicate that aerobic glycolysis is likely suppressed in PEL cells though further investigations are required to understand the metabolic reprogramming in the PEL cells, as well as how it might affect cell proliferation and survival.

## Discussion

**Fig 11:**
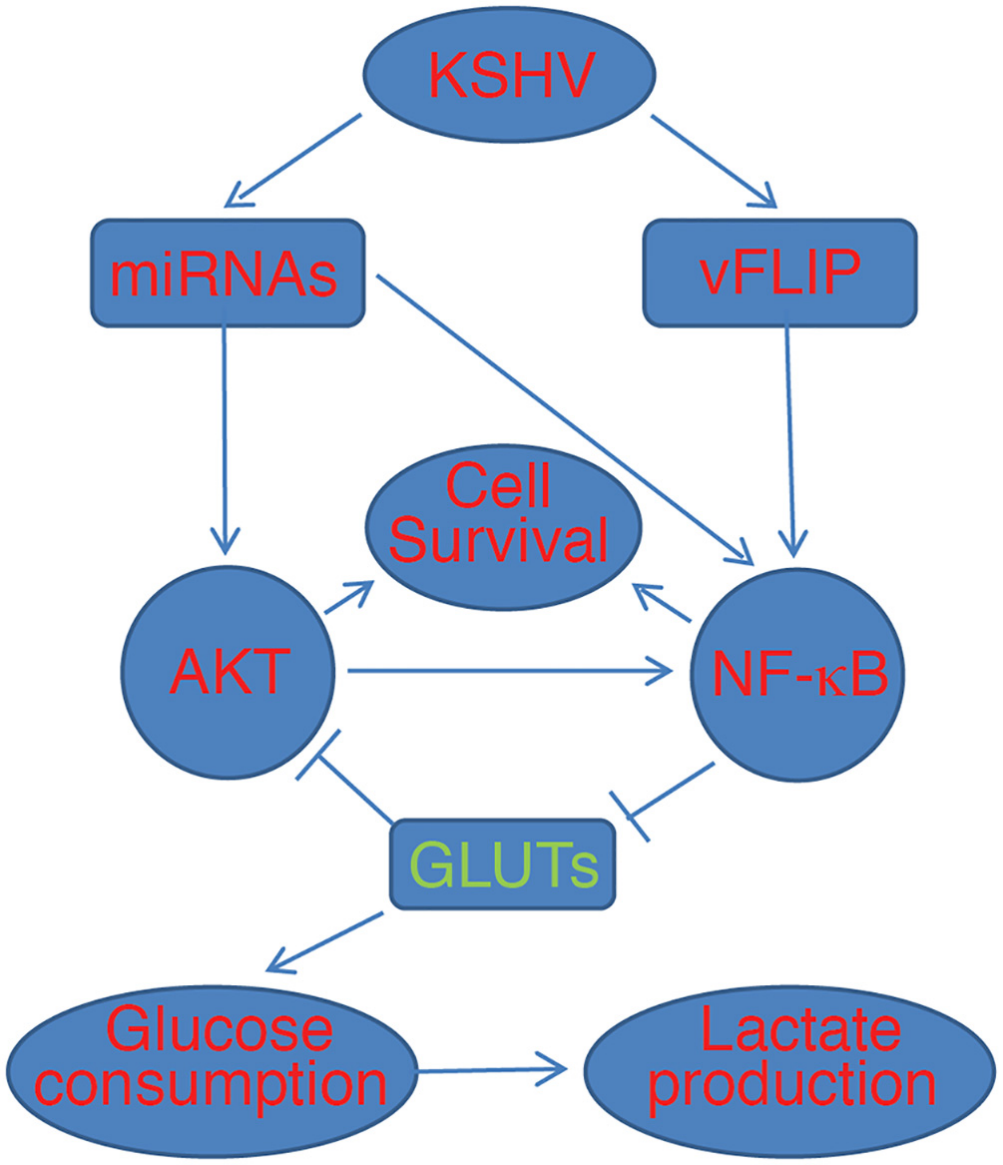
A model illustrates that vFLIP and the miRNA cluster mediate KSHV suppression of aerobic glycolysis to promote cell survival by downregulating the expression of GLUT1 and GLUT3 in an AKT-NF-κB-dependent manner.

We have shown that KSHV downregulates the expression of GLUT1 and GLUT3 to inhibit glucose uptake resulting in the suppression of aerobic glycolysis and oxidative phosphorylation. Under glucose deprivation condition, downregulation of GLUT1 and GLUT3 is required for optimal cell survival and efficient colony formation of KSHV-transformed cells in softagar. Significantly, we have detected downregulation of GLUT1 and GLUT3 in KSHV-infected cells in KS tumors, suggesting that suppression of aerobic glycolysis is likely important in these tumors. Mechanistically, KSHV inhibits the expression of GLUT1 and GLUT3 through activation of the NF-κB pathway by vFLIP and the miRNA cluster. Downregulation of GLUT1 and GLUT3 further maximizes KSHV activation of the AKT-NF-κB survival pathway resulting in enhanced cell survival and cellular transformation. These results have also revealed a negative feedback loop of the AKT-NF-κB pathway imposed by the glucose transporters, which is disrupted by vFLIP and the miRNA cluster (Fig 11).

To adapt to diverse conditions for growth, proliferation and survival, cancer cells must undergo reprograming of the metabolic pathways[4]. For fast growing cancer cells, glucose is diverted to aerobic glycolysis from the TCA cycle and oxidative phosphorylation to provide rapid supply of the energy and substrates for synthesis of macromolecules[3]. Surprisingly, we have found that both aerobic glycolysis and oxidative phosphorylation are suppressed in KSHV-transformed cells. It has been recognized that metabolic pathways must be tightly regulated to ensure the homeostasis of cells, particularly under stress conditions as overflow of the metabolic pathways could generate cytotoxic products[4]. Mesenchymal stem cells have an intrinsic high level of aerobic glycolysis compared to differentiated cells[24]. Thus, suppression of the glycolytic and oxidative phosphorylation activities by KSHV might avoid the overflow the pathways. Interestingly, it has been reported that aerobic glycolysis is upregulated in untransformed KSHV-infected ECs compared to the uninfected control cells[13, 16]. AKT hyperactivation by KSHV is responsible for GLUT1 membrane exposure in KSHV latent infection of a human monocytic cell line[25]. Whether these contradictory observations are due to different cell types or the states of cellular transformation remains to be determined.

The origin of KS tumor cells remains unclear. Our previous studies indicate that KS tumor cells could be derived from mesenchymal stem cells[9, 10]. In the KMM model, KSHV-induced cellular transformation is immediate upon KSHV infection and is dependent on the KSHV genome[8]. If this scenario exists in human KS tumors, downregulation of GLUT1 and GLUT3, and suppression of aerobic glycolysis should be readily present regardless of the status of acute or persistent infection in the tumors. In contrast, if KS tumor cells are derived from endothelial cells, enhanced aerobic glycolysis without suppression of GLUT1 and GLUT3 should be expected. However, our results so far indicate that GLUT1 and GLUT3 are downregulated in KS tumors (Fig 9). It remains possible that KS tumor cells are derived from endothelial cells and are transformed by KSHV in KS tumors but the cellular transformation phenotype has not been genuinely recapitulated in any of cell culture systems, which could explain the discrepancies between the in vivo and in vitro phenotypes. Further studies are required to clarify these contradictions.

The Warburg effect in a tumor is often measured by the avidity of fluorodeoxyglucose (FDG), a glucose analog. Low FDG avidity was detected in pulmonary and lymph node KS but not in skin KS[26, 27]. However, occult KS lesions were detected by FDG-positron emission tomography and computed tomography (FDG-PET/CT) in advanced KS[28, 29]. It was also reported that 55% (5 in 9) of KSHV-associated MCD patients who had cutaneous KS showed mildly hypermetabolic cutaneous abnormalities in FDG-PET[30]. Therefore, whether KS tumors, particularly early stage KS tumors, have increased glucose uptake remains unclear. It should be noted that KS lesions are highly heterogeneous, consisting of LANA-positive spindle tumor cells, and various LANA-negative cell types including vascular and lymphatic endothelial cells, macrophages, lymphocytes, plasma cells and red blood cells[31]. Recent studies have shown that some cancer cells have low levels of aerobic glycolysis but they induce aerobic glycolysis in neighboring stromal cells, which in turn provide fuels for the cancer cells and contribute to the overall Warburg effect in the tumors[32–34]. This model, termed “reverse Warburg effect”, explains some challenges of the Warburg effect and reveals the complex metabolic interactions of tumor and tumor microenvironments. The detection of Warburg effect in a fraction of the KS tumors could also be a reflection of the aerobic glycolytic activities of the stromal cells rather than the LANA-positive tumor cells. In fact, our results have clearly shown that the LANA-positive tumor cells express lower levels of GLUT1 and GLUT3 than the LANA-negative cells. Furthermore, advanced KS tumors are often composed of diverse genetic alterations, some of which could result in metabolic reprograming in these cells. Further research is warranted to delineate the molecular basis underlying the metabolic heterogeneity in KS tumors.

The findings that KSHV inhibits aerobic glycolysis are analogous to results of several studies on PKM2. As a tumor-specific glycolytic enzyme, PKM2 promotes the proliferation of cancer cells by inhibiting ATP generation and antagonizing the Warburg effect in some cancers[35–38]. This observation was initially regarded as counterintuitive but it has become clear that the shift of glucose to the TCA cycle and oxidative phosphorylation can generate metabolic intermediates for the synthesis of lipids, nucleotides and amino acids in addition to ATP[4, 39]. On the other hand, compared to cancer cells that have upregulated levels of PKM2, KSHV-transformed cells are distinct in that they have lower levels of intracellular ATP and oxygen consumption, reflecting the general lower activities of the TCA cycle and oxidative phosphorylation. Since hyperactivation of oxidative phosphorylation can generate reactive oxygen species[6], minimizing ATP production and oxygen consumption might allow KSHV-transformed cells to maintain a balanced cellular redox status.

The fact that KSHV-transformed cells consume less glucose than the untransformed cells despite their faster proliferation rates implies that they might utilize other carbon sources to support their proliferation. Recent studies have shown that in addition to glucose, cancer cells also utilize glutamine, one carbon amino acids or fatty acids to support their growth[4, 5]. Indeed, PEL cells and KSHV-infected ECs utilize fatty acids to support their proliferation and survival[14, 15]. Whether KSHV-transformed cells also depend on these carbon sources for proliferation remains to be investigated. Regardless the alternative carbon sources, such metabolic reprogramming enables KSHV-transformed cells to adapt to glucose-deprived condition. We have shown that under this condition, KSHV-transformed cells maintain normal proliferation and cellular transformation while the untransformed cells undergo arrest and apoptosis. Cancer cells, particularly those in solid tumors, often encounter stress conditions including nutrient deprivation[40, 41]. Glucose concentrations are frequently 3- to 10-fold lower in tumors than in normal tissues[40, 41]. Thus, the observed metabolic reprograming provides the advantage for KSHV-transformed cells to survive in a stress tumor microenvironment. These findings are consistent with results of another study showing that deficiency in PKCξ promotes the plasticity necessary for cancer cells to survive and proliferate in the absence of glucose by reprograming their metabolism[42]. In fact, up to 30% of cancers are FDG-PET-negative, indicating the lack of excessive glucose consumption in these cancers[43]. A number of cancers can survive therapies aimed at curtailing the supply or utilization of glucose by reprogramming their metabolic needs[44, 45]. As a result, such treatment often leads to increasing cancer aggressiveness[44, 45]. It is important to note that, under normal culture condition, KSHV-transformed cells are capable of consuming glucose, and maintaining aerobic glycolytic and oxidative phosphorylation activities albeit at lower levels than the untransformed cells. Thus, KSHV-transformed cells likely have optimized their metabolic pathways to adapt to different proliferation conditions.

Our results show that both KSHV vFLIP and the miRNA cluster are required for suppressing GLUT1 and GLUT3 expression by activating the NF-κB pathway. While overexpression of vFLIP or the miRNA cluster is sufficient to activate the NF-κB pathway[21–23], both are required for the maximal activation of the pathway in KSHV-transformed cells[10]. The mechanism by which vFLIP and the miRNA cluster synergize with each other to maximize the activation of the NF-κB pathway remains unclear.

The NF-κB pathway transduces crucial survival signals and is frequently activated in cancer. GLUT3 is a NF-κB transcriptional target[46–48] and RelA inactivation can lead to upregulation of GLUT1 and GLUT3[48]. Silencing of RelA in murine tumors that heavily rely on NF-κB activation resulted in increased activity of aerobic glycolysis, rendering these tumors especially sensitive to metabolic challenges including glucose deprivation[48]. Indeed, silencing RelA or inhibition of the NF-κB pathway leads to upregulation of GLUT1 and GLUT3, and increase of aerobic glycolysis in KSHV-transformed cells (Figs 5 and 6). These results illustrate NF-κB as a central regulator of energy homeostasis and metabolic adaptation in addition to its pro-survival function. Importantly, NF-κB activation by KSHV miRNAs is essential for the survival, proliferation and cellular transformation[10]. Similarly, vFLIP is also required for KSHV-induced cellular transformation (Fig 3J). Thus, by activating the NF-κB pathway, both KSHV vFLIP and the miRNA cluster play critical roles in KSHV-induced cellular transformation by regulating energy homeostasis and metabolic adaptation in addition to providing survival signal. Interestingly, overexpression of GLUT1 and GLUT3 suppresses NF-κB activation (Fig 8A). Thus, higher levels of GLUT1 and GLUT3 might suppress the NF-κB pathway in primary cells. By activating the NF-κB pathway, KSHV vFLIP and the miRNA cluster inhibit the expression of GLUT1 and GLUT3 in KSHV-transformed cells, which further enhance the AKT and NF-κB signaling. These results have established a NF-κB signaling loop negatively regulated by the glucose transporters, which is disrupted by KSHV vFLIP and the miRNA cluster (Fig 11).

The PI3K/AKT pathway is often hyperactivated in malignant cells and is known to promote the survival and proliferation of cancer cells[49]. We have shown that KSHV-transformed cells have hyperactivated AKT. Both KSHV GPCR (ORF74) and ORF-K1 can activate the AKT pathway[50–52]; however, KSHV-transformed cells are predominantly latent with minimal expression of these two viral proteins[8]. Both cellular and viral IL-6 can also activate the AKT pathway through the gp130 receptor[53, 54]. We have shown that stable overexpression of GLUT1 or GLUT3 suppresses AKT activation. Thus, KSHV downregulation of GLUT1 and GLUT3 can maximize AKT activation. While increased glucose uptake is known to enhance AKT activation[55, 56], the roles of glucose transporters in AKT activation are unclear. Our results indicate that glucose metabolism and glucose transporters might regulate AKT signaling by distinct mechanisms.

AKT is an important driver of the tumor glycolytic phenotype and stimulates ATP generation through multiple mechanisms[57, 58]. In particular, AKT1 simulates aerobic glycolysis by promoting the transcription and incorporation of GLUT1 into the plasma membrane[59–61]. Thus, suppression of aerobic glycolysis in KSHV-transformed cells where there is hyperactivation of AKT appears to contradict with these observations; however, this might be due to the intrinsic high level of aerobic glycolysis in the mesenchymal stem cells[24]. Similarly, hypoxia and HIF1α also enhance aerobic glycolysis[4, 5], and HIF1α is upregulated in KSHV-infected cells[62]. It is possible that NF-κB is the dominant pathway that regulates the expression of GLUT1 and GLUT3, and aerobic glycolysis in KSHV-transformed cells though further investigations are required to clarify these issues.

AKT is a known upstream regulator of NF-κB[49]. Indeed, chemical inhibition of the AKT pathway reduces the level of activated NF-κB, and sensitizes KSHV-transformed cells to glucose deprivation (Fig 8E and 8F). However, the activated AKT only partially accounts for the NF-κB activities as activation of the NF-κB pathway by both vFLIP and miRNAs is independent of the AKT pathway[21–23]. Nevertheless, AKT hyperactivation is essential for the survival and proliferation of KSHV-transformed cells, particularly under stress conditions such as glucose deprivation, and KSHV downregulation of GLUT1 and GLUT3 can maximize the AKT activation.

In summary, KSHV suppression of aerobic glycolysis and oxidative phosphorylation through inhibition of glucose uptake enables the adaption of KSHV-transformed cells to different proliferation and survival conditions. Our findings illustrate the importance of fine-tuning of the metabolic pathways in cancer cells, which could be explored for therapeutic application.

## Materials and Methods

### Cell Culture and Reagents

Rat primary embryonic metanephric mesenchymal precursor cells (MM), KSHV-transformed MM cells (KMM)[8], MM cells infected by KSHV mutants with a cluster of 10 precursor miRNAs deleted (ΔmiRs)[10], vFLIP deleted (ΔvFLIP)[18], vCyclin deleted (ΔvCyclin)[12] and 293T cells were maintained in Dulbecco’s modified Eagle’s medium (DMEM; with 25 mM glucose, 4 mM L-glutamine and 2 mM sodium pyruvate) supplemented with 10% fetal bovine serum (FBS; Sigma-Aldrich, St. Louis, Mo) and antibiotics (100 μg/mL penicillin and 100 μg/mL streptomycin). Only MM and KMM cells at early passage (<15) were used for the experiments. For glucose starvation, cells were cultured in DMEM without glucose (with 4 mM L-glutamine and 2 mM sodium pyruvate), supplemented with 10% FBS (Sigma-Aldrich). PEL cell lines BCBL1, BC3 and BCP1, and EBV-negative Burkitt’s lymphoma cell line BJAB and KSHV-infected BJAB (BJAB-KSHV) were cultured in RPMI-1640 medium with 10% FBS. JSH-23 (inhibitor of NF-κB nuclear translocation), BAY 11–7082 (an inhibitor of IκBα phosphorylation) and LY294002 (PI3K inhibitor) were purchased from Sigma-Aldrich.

### Cell Proliferation and Softagar Colony Assays

Softagar assay was performed as previously described[12]. Briefly, a total of 2x10^4^ cells suspended in 1 ml of 0.3% top agar (Cat. A5431, Sigma-Aldrich) were plated onto one well of 0.5% base agar in 6 well-plates and maintained for 2–3 weeks. Colonies with a diameter of >50 μm were counted and photographed at 40× magnification using a microscope.

### Cell Cycle Analysis, Apoptosis Assay and BrdU Incorporation

Cell cycle and BrdU incorporation were performed at the indicated time points as previously described[12]. Cell cycle was analyzed by propidium iodide (PI) staining. BrdU incorporation was performed by pulsing cells with 10 μM BrdU for 1 h and then stained with a Pacific Blue monoclonal antibody to BrdU (Cat. B35129, Life Technologies, Grand Island, NY). Apoptotic cells were detected by Fixable Viability Dye eFluor 660 staining (Cat. 650864, eBioscience, San Diego, CA) and with a PE-Cy7 Annexin V Apoptosis Detection Set (Cat. 88810374, eBioscience) following the instructions of the manufacturer. Flow cytometry was performed in a FACSCanto System (BD Biosciences, San Jose, CA) and analysis was performed with FlowJo (FlowJo, LLC, Ashland, OR).

### Measurements of Glucose, Lactate, ATP and Oxygen

Cells were seeded in 24-well plates, media were changed 24 h later and assays were carried out at the indicated time points in normal medium or in glucose-free medium (glucose starvation). Glucose and lactate concentrations were measured in the culture media using the Glucose Colorimetric/Fluorometric Assay Kit (Cat. K606-100, BioVision, Milpitas, CA) and the Lactate Assay Kit (Cat. MAK064, Sigma-Aldrich), respectively, according to the manufacturer’s instructions. Intracellular ATP levels were determined in cell lysates using the ATP Bioluminescent Somatic Cell Assay Kit (Cat. FLASC-1KT, Sigma-Aldrich) according to the manufacturer’s instructions. Oxygen uptake was measured in 24-well plates using a Seahorse XF24 Extracellular Flux Analyzer (Seahorse Bioscience, Billerica, MA). Cells were seeded at 2x10^4^ cells per well and incubated overnight in normal growth medium. Next day, medium was changed to 8.3 g/L DMEM base medium at pH 7.4 supplemented with 200 mM GlutaMax-1, 100 mM sodium pyruvate and 32 mM NaCl in the presence of 25 mM glucose, and oxygen consumption was continuously measured.

### Lentiviral Vectors and Lentiviral Infections

The coding sequences of rat GLUT1 (GenBank accession no. NM_138827.1) and GLUT3 (NM_017102.2) were cloned into the FseI/PacI sites of pSMPUW-IRES-Bsd (Cat.VPK-219, Cell Biolabs, San Diego, CA) by PCR amplification to generate expression vectors named pSMPUW-IRES-Bsd-GLUT1 and pSMPUW-IRES-Bsd-GLUT3. The primers were 5’-AGTGGCCGGCCATGGAGCCCAGCAGCAAGA-3’ (forward) and 5’-AGTTTAATTAATCACACTTGGGAGTCAGCC-3’ (reverse) for GLUT1, and 5’-AGTGGCCGGCCATGGGGACAGCGAAGGTGA-3’ (forward) and 5’-AGTTTAATTAATCAGGCATTGCCAGGGGTCT-3’ (reverse) for GLUT3 with all the restriction enzyme sites underlined. All constructs were confirmed by direct DNA sequencing. The mCherry coding sequence from the pmCherry-C1 vector (Addgene, Cambridge, MA) and the rat LC3 coding sequence from the pEGFP-LC3 vector (Addgene) were cloned into the XbaI/BamHI and BamHI/NotI sites of the pCDH-hygro vector to generate a fusion mCherry-LC3 expression vector named pCDH-mCherry-(rat)LC3. To obtain the recombinant lentivirus, pSMPUW-IRES-Bsd overexpression plasmids were cotransfected with pMDLg/pRRE, pRSV-Rev and pMD2.G packaging plasmids into actively growing HEK293T cells by using Lipofectamine 2000 transfection reagent. Virus-containing supernatants were collected 72 hr after transfection and filtered to remove cells, and target cells were infected in the presence of 8 μg/mL polybrene. MM-Vector, KMM-Vector, MM-GLUT1, KMM-GLUT1, MM-GLUT3 and KMM-GLUT3 cells were selected with 10 μg/mL Blasticidin after transduction.

### siRNA Knock Down of RelA

siRNA knock down of RelA was performed as previously described[10]. Briefly, the small interfering (si)RNA targeting Rat RelA (GenBank Access. No. NM_199267.2) transcript was designated siRelA (sense: GUGACAAAGUGCAGAAAGAUU; antisense: UCUUUCUGCACUUUGUCACUU). A scrambled oligonucleotide containing a random sequence was obtained from the manufacturer (Ambion, Thermo Fisher Scientific, Waltham, MA) and used as a control. Reverse transfection of siRNA duplex was performed using Lipofectamine-RNAiMAX (Invitrogen, Carlsbad, CA). Transfection was performed at a final concentration of 50 nM.

### Reverse Transcription Quantitative Real-Time Polymerase-Chain Reaction (RT-qPCR)

Total RNA was isolated with TRI Reagent (Cat. T9424, Sigma) according to the instructions of the manufacturer. Reverse transcription was performed with total RNA using Maxima H Minus First Strand cDNA Synthesis Kit (Cat. K1652, Thermo Fisher Scientific). qPCR analysis was performed on Eppendorf Real Plex using KAPA SYBR FAST qPCR Kits (Cat. KK4602, Kapa Biosystems, Wilmington, MA). The relative expression levels of target genes were normalized to the expression of internal control genes, which yielded a 2^-ΔΔCt^ value. All reactions were run in triplicates. The cycle threshold (Ct) values should not differ more than 0.5 among triplicates. Rat β-actin was used as an internal control. The primers were 5’-GCGAGCTCTTTGAATGTGTG-3’ (forward) and 5’-GGCTCAGGTCCTTCACGTAG-3’ (reverse) for GLUT1, 5’-ATGTTGGCCAGTCAAGTTCC-3’ (forward) and 5’-CTGTCACCTCTGGGAGCAG-3’ (reverse) for GLUT3, and 5’-GCAGGAGTACGATGAGTCCG-3’ (forward) and 5’-ACGCAGCTCAGTAACAGTCC-3’ (reverse) for β-actin.

### Western-Blot Analysis

Total cell lysates were separated in SDS-polyacrylamide gels, electrophoretically transferred to nitrocellulose membranes (GE Healthcare, Piscataway, NJ). The membranes were incubated sequentially with primary and secondary antibodies. The signal was developed using Luminiata Crescendo Western HRP substrate (cat. WBLUR0500, EMD Millipore, Billerica, MA). The antibodies used for Western blot include rabbit monoclonal antibodies (mAbs) for GLUT1 (cat. ab115730, Abcam, Cambridge, MA), phospho-AKT (Thr308) (cat. 2965, Cell Signaling Technology, Danvers, MA) and NF-κB p65 (cat. 8242, Cell Signaling Technology); rabbit polyclonal antibodies against GLUT3 (cat. ab41525, Abcam), phospho-4E-BP1 (Ser65) (cat. 9451, Cell Signaling Technology) and AKT (cat. 9272, Cell Signaling Technology); and mouse mAbs for LC3 (cat. CTB-LC3-1-50, COSMO BIO CO., Tokyo, Japan), phospho-NF-κB p65 (Ser 536) (cat. 3036, Cell Signaling Technology) and β-tubulin (7B9, Sigma).

### Flow Cytometry

Cells fixed with 80% methanol (5 min) were permeabilized with 0.1% PBS-Tween for 20 min. The cells were then incubated in PBS containing 10% normal goat serum and 0.3 M glycine to block non-specific protein-protein interactions followed by GLUT1 or GLUT3 antibody (ab115730 or ab41525, respectively) at 1/500 dilution for 30 min at room temperature. The secondary antibody used was Alexa conjugated at 1/2000 dilution for 30 min at room temperature. Flow cytometry was performed with a FACS Canto II flow cytometer and analyzed with FlowJo. All runs included a control without the primary antibody.

### Confocal Fluorescence Microscopy

KMM, KMM-GLUT1 and KMM-GLUT3 cells were seeded on 24-well culture plate that contained 12 mm diameter round glass coverslips (2x10^4^ cells per well). After infection 48 h, cells were fixed with 4% paraformaldehyde in PBS for 15 min at room temperature and washed with PBS. Samples were incubated with 0.5 μg/ mL 4-, 6-diamidino-2-phenylindole (DAPI) in PBS for 1min, then were mounted in FluorSave Reagent (Calbiochem, San Diego, CA). Samples were imaged with laser-scanning confocal microscopy (Nikon Eclipse C1).

### Immunofluorescence Staining of Tissue Sections

Formalin-fixed, paraffin-embedded tissue microarray consisting of tissue specimens from healthy subjects and patients with KS were obtained from the AIDS and Cancer Specimen Resource (ACSR). Sections were de-paraffinized in xylene, rehydrated through graded ethanol, quenched for endogenous peroxidase activity in 3% hydrogen peroxide in methanol for 10 min and processed for antigen retrieval by microwave heating in 1 mM EDTA at pH 8.0. Immunostaining was performed using an anti-LANA antibody LN35 (cat. ab4103, Abcam) and an anti-GLUT1 antibody (ab115730, Abcam) or an anti-GLUT3 antibody (cat. sc-30107, Santa Cruz Biotechnology, Santa Cruz, CA) antibodies, followed by Alexa-488 and Alexa-568 conjugated secondary antibodies (Thermo Fisher Scientific). Nuclei were stained with 4’,6-diamidino-2-phenylindole (DAPI). The stained cells were viewed under a confocal fluorescence microscope with a 60x objective. Tissue sections without incubating with primary antibody were used as negative controls. For each specimen, three images of representative areas were acquired and a total of 200 to 500 cells were counted unless stated otherwise. The scoring of the expression of GLUT1 and GLUT3 was performed using a modified Histo-score (H-score), which included a semi-quantitative assessment of both fraction of positive cells and intensity of staining. The intensity score was defined as no staining (0), and weak (1), moderate (2), or strong (3) staining. The fraction score was based on the proportion of positively stained cells (0%-100%). The intensity and fraction scores were then multiplied to obtain H-score, which ranged from 0 to 3 and represented the levels of GLUT1 and GLUT3 expression.

### Statistics

Data were expressed as the mean ± standard error of the mean (s.e.m.) from at least three independent experiments, each with three repeats unless stated otherwise. The differences between groups were analyzed using Student’s t-test when two groups were compared and using one-way ANOVA when more than two groups were compared unless otherwise noted. Correlation was determined using Spearman’s correlation coefficient. Statistical tests were two-sided. A P < 0.05 was considered statistically significant. Statistical symbols “*”, “**” and “***” represent P-values < 0.05, < 0.01 and < 0.001, respectively, while “NS” indicates “not significant”. All analyses were performed using the GraphPad Prism program (GraphPad Software Inc., San Diego, CA).

## Supporting Information

S1 Fig Expression alterations of enzymes in different steps of glycolysis pathway. The numbers shown were the ratios of mRNA expression levels of the enzymes in KMM cells over those of MM cells[10].
(TIF)

S2 Fig GLUT1 expression is downregulated in KSHV infected cells in human KS tumors. Expression of GLUT1 was quantified based on immunofluorescence staining in human KS tissues (n = 25) and normal skin tissues (n = 2), using a modified His-score as described in the Materials and Methods. For KS tissues, the differences between LANA-negative (-) and LANA-positive (+) cells were performed by Wilcoxon matched-pairs signed-ranks test. *P < 0.05; **P < 0.01; ***P < 0.001; NS, not significant.
(TIF)

S3 Fig GLUT3 expression is downregulated in KSHV infected cells in human KS tumors. Expression of GLUT3 was quantified based on immunofluorescence staining in human KS tissues (n = 19) and normal skin tissues (n = 3), using a modified His-score as described in the Materials and Methods. For KS tissues, the differences between LANA-negative (-) and LANA-positive (+) cells were performed by Wilcoxon matched-pairs signed-ranks test. *P < 0.05; ***P < 0.001; NS, not significant.
(TIF)
