# Supplementary figures and images for: An Oncogenic Virus Promotes Cell Survival and Cellular Transformation by Suppressing Glycolysis
Source: PLoS Pathog. 2016 May 17;12(5):e1005648. doi: 10.1371/journal.ppat.1005648 (PMC4871371; doi:10.1371/journal.ppat.1005648)

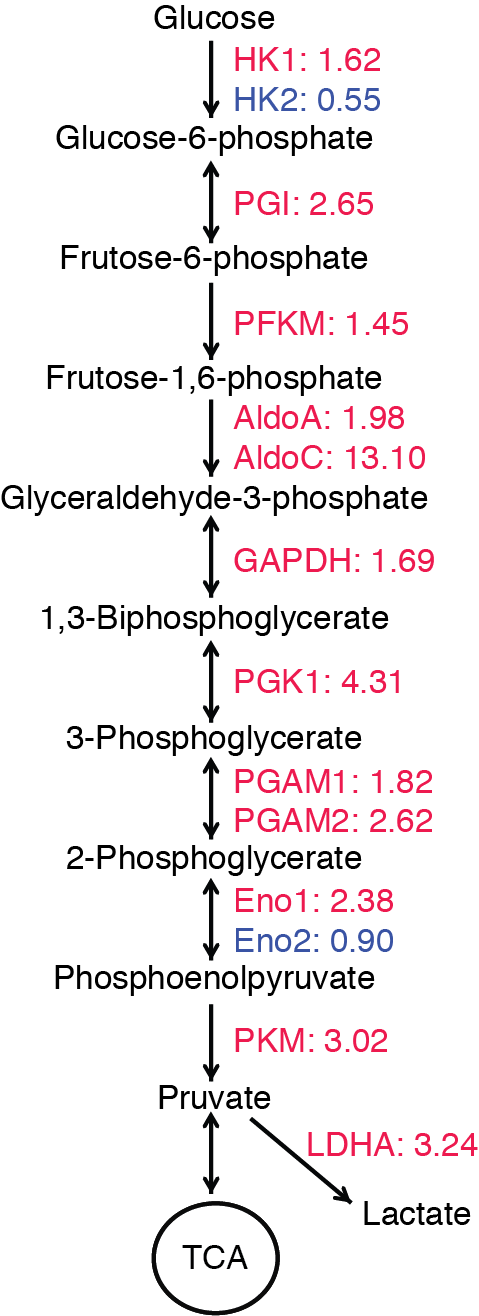

Supplement: S1 Fig — The numbers shown were the ratios of mRNA expression levels of the enzymes in KMM cells over those of MM cells[10]. (TIF) [file ppat.1005648.s001.tif]

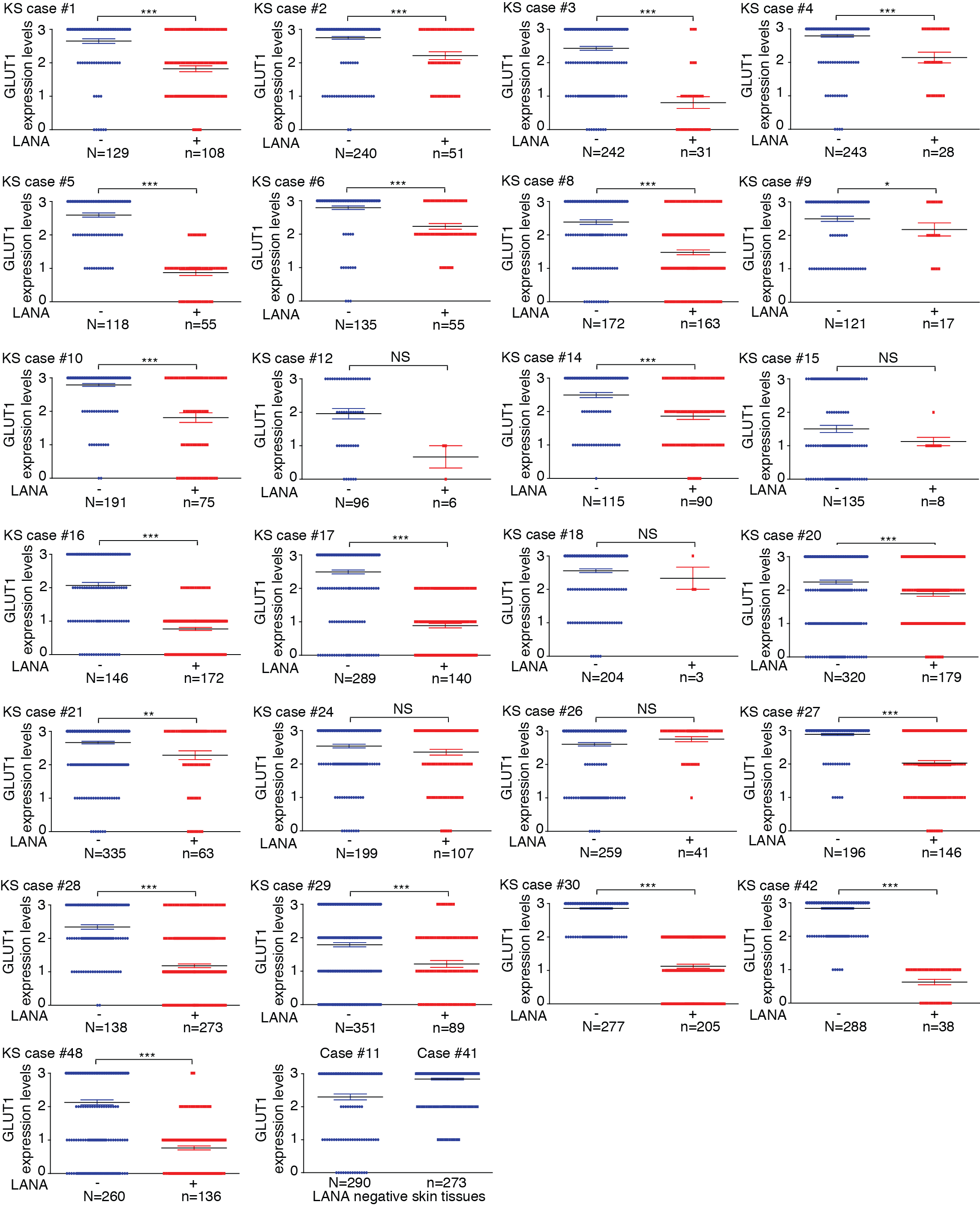

Supplement: S2 Fig — Expression of GLUT1 was quantified based on immunofluorescence staining in human KS tissues (n = 25) and normal skin tissues (n = 2), using a modified His-score as described in the Materials and Methods. For KS tissues, the differences between LANA-negative (-) and LANA-positive (+) cells were performed by Wilcoxon matched-pairs signed-ranks test. *P < 0.05; **P < 0.01; ***P < 0.001; NS, not significant. (TIF) [file ppat.1005648.s002.tif]

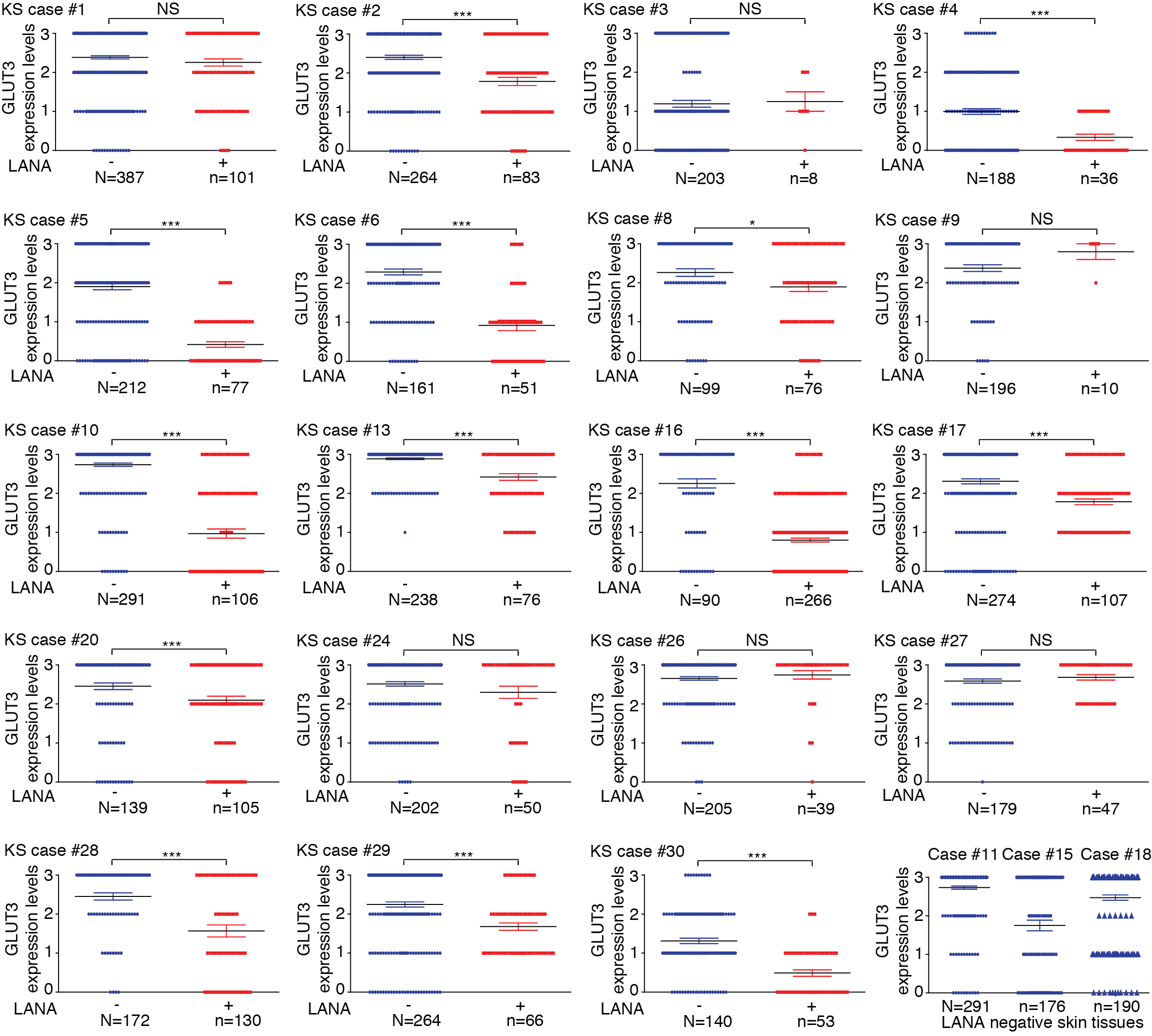

Supplement: S3 Fig — Expression of GLUT3 was quantified based on immunofluorescence staining in human KS tissues (n = 19) and normal skin tissues (n = 3), using a modified His-score as described in the Materials and Methods. For KS tissues, the differences between LANA-negative (-) and LANA-positive (+) cells were performed by Wilcoxon matched-pairs signed-ranks test. *P < 0.05; ***P < 0.001; NS, not significant. (TIF) [file ppat.1005648.s003.tif]
